# Discovery of monocarbonyl curcumin-BTP hybrids as STAT3 inhibitors for drug-sensitive and drug-resistant breast cancer therapy

**DOI:** 10.1038/srep46352

**Published:** 2017-04-11

**Authors:** Wenda Zhang, Jianpeng Guo, Shanshan Li, Ting Ma, Dingqiao Xu, Chao Han, Feiyan Liu, Wenying Yu, Lingyi Kong

**Affiliations:** 1 State Key Laboratory of Natural Medicines, Department of Natural Medicinal Chemistry, China Pharmaceutical University, 24 Tong Jia Xiang, Nanjing 210009, China

## Abstract

Signal transducer and activator of transcription 3 (STAT3) is a well-known antitumor target. Exogenous ROS insult can lead to selective cytotoxicity against cancer cells. A combination of STAT3 inhibition and “oxidation therapy” may be a new strategy to address the multidrug-resistance issue due to their important roles in the survival and drug resistance of cancer cells. Here, a series of novel curcumin-BTP hybrids were designed and evaluated as STAT3 inhibitors with ROS production activity. Compound **6b** exerted the best antitumor activity and selectivity for MCF-7 and MCF-7/DOX cells (IC_50_ = 0.52 μM and 0.40 μM, respectively), while its IC_50_ value for MCF-10A breast epithelial cells was 7.72 μM. Furthermore, compound **6b** suppressed STAT3 phosphorylation, nuclear translocation and DNA-binding activity and the expression of STAT3 specific oncogenes. Increases in the level of IL-6-induced p-STAT3 were also inhibited by **6b** without influencing IFN-γ-induced p-STAT1 expression. Additionally, **6b** effectively promoted intracellular ROS accumulation, induced cancer cell apoptosis and cell cycle arrest, abolished the colony formation ability of breast cancer cells, and inhibited P-gp expression in MCF-7/DOX cells. Finally, **6b** suppressed the growth of implanted human breast cancer *in vivo*. Our findings highlight that **6b** may be a promising therapeutic agent for drug-sensitive and drug-resistant breast cancers.

Breast cancer is the second most fatal cancer in women. The prognosis is generally regarded as poor and the overall survival rate of all stages is less than 1% at 5 years^1^. The rapid emergence of drug resistance also limits the benefits of treatment of this disease^2^. Therefore, it is imperative to develop more effective treatments for breast cancer.

Signal transducer and activator of transcription 3 (STAT3) is a type of latent cytosolic transcription factor^3^,^4^,^5^,^6^,^7^,^8^. Persistent activation of the STAT3 signaling pathway has been documented in a wide range of solid cancers and some drug-resistant cancers in humans and is commonly associated with a worse prognosis^2^,^9^,^10^. Interference with the STAT3 signaling pathway in cancer cells has been shown to result in growth inhibition and the induction of apoptosis, making it an attractive target for cancer therapies^11^,^12^,^13^,^14^,^15^,^16^,^17^,^18^,^19^. Moreover, STAT3 inhibition is a viable treatment option for drug-resistant cancers through inhibiting STAT3-mediated MDR1 gene expression^2^.

Cancer cells are usually exposed to a moderate level of reactive oxygen species (ROS), primarily due to their active metabolism in response to oncogenic signals^20^. In fact, moderate oxidative stress is conducive to several important processes, such as proliferation, angiogenesis, and metastasis, in cancer cells^21^. Nevertheless, high levels of ROS irreversibly damage DNA and lipids and ultimately cause cancer cell apoptosis^22^. An exogenous ROS insult is tolerable to normal cells, but it may exceed the threshold that cancer cells can tolerate and thus lead to selective cytotoxicity against cancer cells. The pharmacological elevation of intracellular ROS has emerged as an effective strategy for selectively targeting cancer cells^23^,^24^, which was considered as “oxidation therapy”^25^,^26^,^27^.

In 2011, Raj, L. *et al*. reported that piperlongumine selectively kills cancer cells by targeting their stress response to ROS^24^. In 2014, Bharadwaj, U. *et al*. reported that piperlongumine is a direct STAT3 inhibitor with potent activity against breast cancer^28^. The inhibition of STAT3 and increase in ROS production are antitumor activities that jointly promote cancer cell apoptosis.

Curcumin is a bioactive component in Curcuma longa and the subject of extensive research due to its relative safety in large dosages. The complex chemistry of curcumin allows it to inhibit multiple oncogenic processes, including those associated with the JAK2/STAT3 pathway or the induction of ROS production^29^,^30^,^31^. To improve the potency of curcumin as a cancer therapeutic agent, many synthetic curcumin analogues have been developed as new JAK2/STAT3 inhibitors, including FLLL31, FLLL32 and FLL62^17^,^18^,^19^. These curcumin-based compounds improve the bioavailability and anticancer potential by stabilizing the central *β*-diketone linker.

The benzo[*b*]thiophene 1, 1-dioxide (BTP) moiety contributes to ROS production and is also a crucial pharmacophore of many STAT3 inhibitors (such as Stattic, HJC0123 and HJC0146). Inhibitors with this moiety significantly inhibit p-STAT3 levels, markedly enhance the ROS level and exhibit anti-proliferative activity in cancer cells^6^,^32^,^33^. Moreover, Stattic, a STAT3 inhibitor containing this moiety, circumvented the cisplatin resistance of an orthotopic xenograft of ovarian cancer *in vivo*. Nevertheless, because of their low activity or poor properties, none of these inhibitors has reached the market yet.

Given that a STAT3 inhibitor with ROS-promoting activity may be a new strategy for the treatment of solid tumors, we hypothesized that these new hybrids of curcumin with a BTP moiety may inhibit STAT3 activation and lead to ROS accumulation and ultimately induce cancer cell apoptosis. To further improve metabolic stability, half of the central linker was stabilized with piperazine, which has an equal length and is widely used in the market drugs (Fig. 1). In this study, twenty-two curcumin-BTP hybrids were designed, synthesized, and biologically evaluated as STAT3 inhibitors with ROS production activity. These new STAT3 inhibitors are promising candidates for breast cancer therapy, due to their drug-like properties and potent bioactivities *in vitro* and *in vivo*.

## Results and Discussion

### Chemistry

The synthetic routes for compounds **6a**–**v** are shown in **Scheme 1** in the supporting information. Compounds **2a** and **2b** were obtained by the reaction of commercially available 3-bromo-2-fluorobenzaldehyde (**1a**) and 4-bromo-2-fluorobenzaldehyde (**1b**), respectively, with methyl thioglycolate. Coupling of **2a** and **2b** with tert-butyl-1-piperazinecarboxylate in the presence of HATU resulted in compounds **3a** and **3b**, respectively, which were then reacted with 3-chlorine peroxide benzoic acid to produce compounds **4a** and **4b**, respectively. Compounds **5a** and **5b** were prepared from **4a** and **4b**, respectively, by deprotecting N-Boc protection with chlorine hydride gas in methanol and dichloromethane (1:1). The desired compounds (**6a**-**6v**) were generated by the coupling of **5a** and **5b** with different cinnamic acids using the same synthetic method used to generate compounds **3a** and **3b**. All the hybrids were confirmed by ^1^H NMR, IR, HRMS (ESI), and ^13^C NMR spectra. The ^1^H NMR spectrums of representative compounds were shown in the S1 of the Supporting Information.

### *In vitro* cell growth inhibition activity

**Figure 2:**
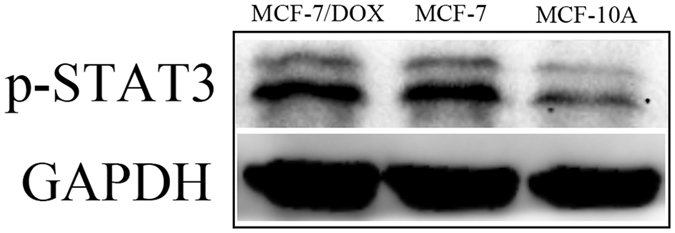
Western blot analysis of p-STAT3 levels in MCF-7, MCF-7/DOX and MCF-10A cells.

Given that compounds **6a**–**v** were designed to target STAT3 in cancer cells, we first examined the levels of p-STAT3 expression in MCF-7, MCF-7/DOX and MCF-10A cells by western blot assays. The levels of p-STAT3 expression in MCF-7 and MCF-7/DOX cells were significantly higher than that in MCF-10A cells (Fig. 2). To determine the structure-activity relationships of the synthetic compounds, MCF-7 and MCF-7/DOX (doxorubicin) cells were treated with the designed compounds and the positive controls, DOX and curcumin, and then their proliferative activity was determined by the MTT assay. The IC_50_ values are summarized in Table 1. Notably, all the compounds exhibited stronger anti-proliferative activity than their mother compound, curcumin. Compound **6b** was the most potent inhibitor of MCF-7 and MCF-7/DOX cell growth with IC_50_ values of 0.52 μM and 0.40 μM, respectively, which was a marked improvement the anti-proliferative activity compared to that of curcumin (IC_50_ = 37.7 μM and 32.7 μM, respectively). In general, the hybrids with a 5-Br substitute on the BTP ring had a slightly stronger activity than the hybrids with a 6-Br substitute. Compounds with electron-withdrawing substitutes such as chlorine (**6h** and **6s**) and fluorine (**6v**) on the benzene ring of the hybrids usually showed less inhibition activity than those with electron-donating substitutes (**6a**, **6b**, **6c**, **6j** and **6u**) with exception of **6i**. Compounds with a methoxy substitute in the para-position of the benzene ring (**6a**, **6b**, **6l**, **6j**, **6m** and **6u**) exerted more potent activity, while replacement of the methoxy group with a hydroxyl group (**6d**, **6o** and **6t**) led to a marked decrease in activity.

Subsequently, the anti-proliferative efficacy of the most potent compound, **6b**, was further investigated using the MTT assay. DOX had similar growth inhibition activity in both tumor and normal cells, while **6b** exhibited a 7- to 21-fold increase in anti-proliferative selectivity for cancer cells than for normal MCF-10A and LO2 cells (IC_50_ = 7.7 μM and 8.3 μM, respectively), suggesting that **6b** may have selective anti-proliferative activity against cancer cells (Table 2).

### Molecular docking

**Figure 3:**
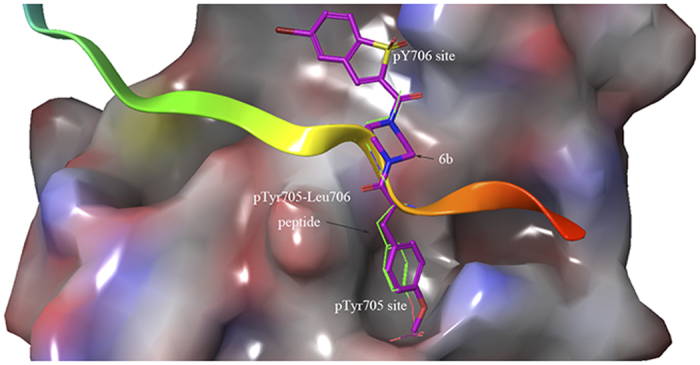
The binding mode of 6b, native pTyr705-Leu706 peptide and STAT3 SH2 domain by AutoDock4.2.

Given that these compounds were designed as STAT3 inhibitors, compound **6b** was used to predict potential binding modes with STAT3 using docking assays. As shown in Fig. 3, **6b** tightly bound to the SH2 domain of STAT3, indicating that **6b** may be a STAT3-SH2 domain inhibitor. The docking mode of **6b** perfectly overlapped with the binding mode of the native pTyr705-Leu706 peptide by forming three hydrogen bonding interactions with the Arg609, Lys626 and Gln635 residues. The predicted binding energy was −8.2 kcal/mol. In addition, the piperazine linker provided a suitable length and angle for the inhibitor to interact with the two binding pockets of STAT3^34^. Together, **6b** was inferred to be a direct STAT3 inhibitor.

### Compound 6b inhibited STAT3 phosphorylation, nuclear translocation and DNA-binding activity in breast cancer cells

**Figure 4:**
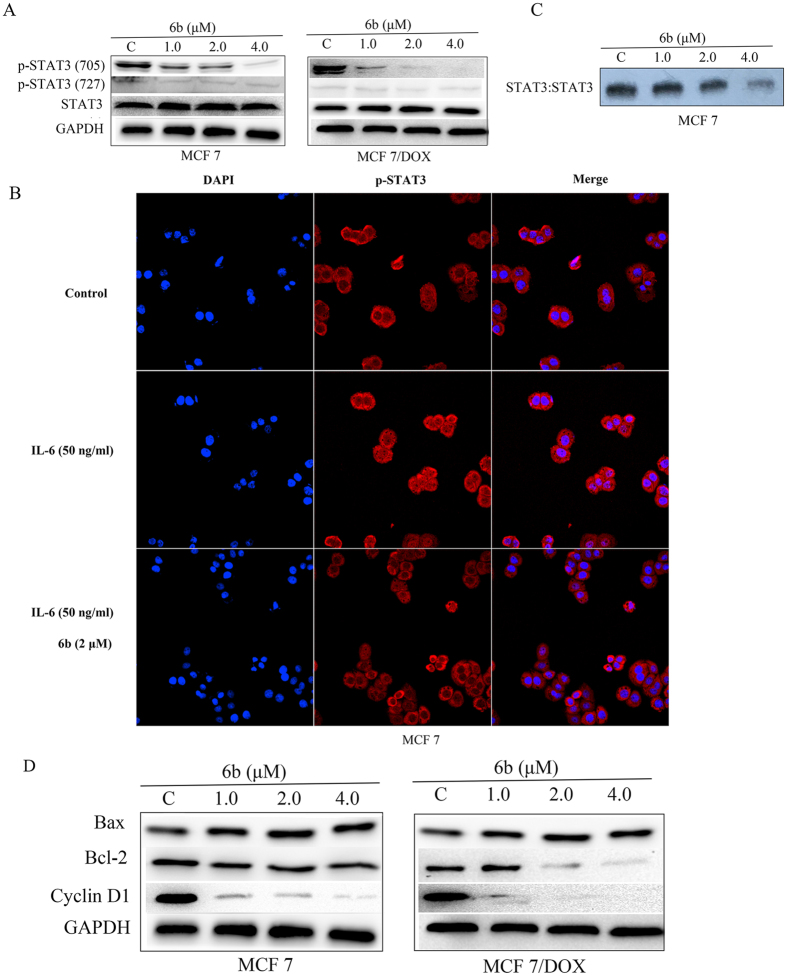
Compound 6b inhibited STAT3 activity. (**A**) Western blot analysis of p-STAT3 (Tyr705), p-STAT3 (Ser727) and STAT3 in whole-cell lysates of equal total protein prepared from MCF-7 and MCF-7/DOX cells treated with compound **6b** for 24 h. (**B**) EMSA analysis of nuclear extracts of equal total protein containing activated STAT3 from MCF-7 cells treated with compound **6b** for 24 h after incubation with hSIE probe that binds STAT3. (**C**) MCF-7 cells were starved overnight followed by treatment with **6b** or DMSO for 6 h. Cells were then treated with 50 ng/ml of IL-6 for 30 min. For immunofluorescent assay, cells were fixed and stained with anti-phospho-STAT3 (p-STAT3) and DAPI before subject to ImageXpress Micro Confocal analysis. Red: p-STAT3; blue: nuclei. (**D**) Western blot analysis of Bcl-2, Bax and Cyclin D1 in MCF-7 and MCF-7/DOX cells treated with compound **6b** for 24 h.

It was reported that targeting the STAT3-SH2 domain can effectively suppress the phosphorylation of STAT3^35^. To determine the role of **6b** as a STAT3 inhibitor, we investigated the effects of **6b** on p-STAT3 (Tyr705) and p-STAT3 (Ser727) protein expression by western blot assays. Exposure of MCF-7 and MCF-7/DOX cells to **6b** led to a dose-dependent inhibition of STAT3 phosphorylation at the pTyr705 residue but had little effect on STAT3 phosphorylation at the Ser727 residue or total STAT3 protein level (Fig. 4A).

Nuclear translocation of STAT3 plays a critical role in STAT3:STAT3/DNA complex formation and its target gene expression, so we further investigated the inhibitory effect of **6b** on preventing STAT3 from translocating to the nucleus. As shown in Fig. 4B, p-STAT3 protein (red) was retained in both the cytoplasm and nucleus in MCF-7 cells not treated with **6b** or IL-6. In MCF-7 cells treated with IL-6 at 50 ng/ml, an increased level of p-STAT3 protein (red) was observed in the nucleus. However, treatment with **6b** led to a sharp decrease of the p-STAT3 level in the nucleus, suggesting that **6b** suppressed STAT3 nuclear translocation.

As a STAT3 inhibitor, **6b** is supposed to prevent dimeric STAT3 from binding to certain DNA sequences in the nucleus. Nuclear extracts of human breast MCF-7 cells treated with **6b** were prepared and subjected to EMSA analysis using an hSIE probe. Compound **6b** strongly inhibited STAT3 DNA-binding activity in a dose-dependent manner (Fig. 4C). Taken together, these results show that **6b** effectively inhibited STAT3 activation.

### Compound 6b regulated STAT3 target oncogenes in breast cancer cells

**Figure 5:**
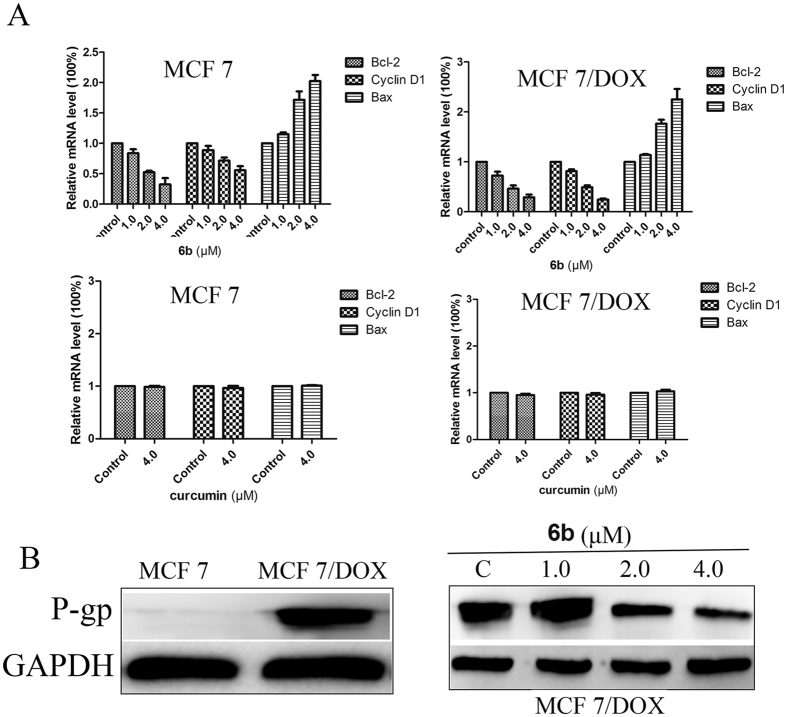
Effects of compound 6b on the STAT3 target genes and the levels of P-gp. (**A**) RT-PCR analysis of Bcl-2, Bax and Cyclin D1 in MCF-7 and MCF-7/DOX cells treated with compound **6b** for 24 h with curcumin as reference. (**B**) Western blot analysis of P-gp levels in whole-cell lysates of equal total protein prepared from MCF-7, MCF-7/DOX cells and MCF-7/DOX cells treated with compound **6b** for 24 h.

We had elucidated that **6b** could effectively inhibit STAT3 phosphorylation, nuclear translocation and DNA-binding activity. To gain more insight into the antitumor activity and mechanistic effects of **6b** on the STAT3 pathway, we examined its effect on STAT3 target genes such as Bcl-2 and Bax, which are related to proliferation, and Cyclin D1, which regulates the cell cycle. MCF-7 and MCF-7/DOX cells were treated with compound **6b** and processed for western blot assays and qRT-PCR analysis. As shown in Fig. 4D, compound **6b** inhibited the expression of Bcl-2 and Cyclin D1 and increased the expression of Bax. Similarly, Bcl-2 and Cyclin D1 gene levels were decreased in a dose-dependent manner and that of Bax was upregulated by **6b**, while treatment with curcumin had little effect on these gene expression levels. The decreases in the gene expression level of Bcl-2 and at 1.0, 2.0, and 4.0 μM concentrations of **6b** were 11.7%, 45.8% and 61.2%, respectively, in MCF-7 cells and 27.5%, 43.5% and 71.0%, respectively, in MCF-7/DOX cells. The decreases in the gene expression level of Cyclin D1 at 1.0, 2.0, and 4.0 μM concentrations of compound **6b** were 16.0%, 28.4% and 45.5%, respectively, in MCF-7 cells and 18.5%, 51.0% and 75.0%, respectively, in MCF-7/DOX cells. Bax gene expression increased by 15.0%, 71.5% and 97.5% in MCF-7 cells and 14.0%, 76.5% and 125.0% in MCF-7/DOX cells when treated with 1.0, 2.0, and 4.0 μM concentrations of compound **6b**, respectively (Fig. 5A).

### Compound 6b inhibited the expression of P-gp in MCF-7/DOX breast cancer cells

Previous studies have shown that overexpression of P-glycoprotein (P-gp) in cancer cells can lead to the export of anticancer drugs and cause consequent drug ineffectiveness, which is a leading factor for tumor multidrug-resistance (MDR) and inhibiting STAT3 activation could effectively decrease P-gp expression^2^,^36^. Thus, to further investigate the role of **6b** in suppressing MCF-7/DOX cell proliferation, we examined the effect of **6b** on P-gp in MCF-7/DOX cells. We determined the levels of P-gp expression in MCF-7 and MCF-7/DOX cells using western blot assays. As shown in Fig. 5B, high levels of P-gp were expressed in MCF-7/DOX cells while low levels of P-gp were detected in MCF-7 cells. Compound **6b** decreased P-gp expression in MCF-7/DOX cells in a dose-dependent manner.

### Compound 6b induced breast cancer cell apoptosis and inhibited breast cancer cell colony formation

**Figure 6:**
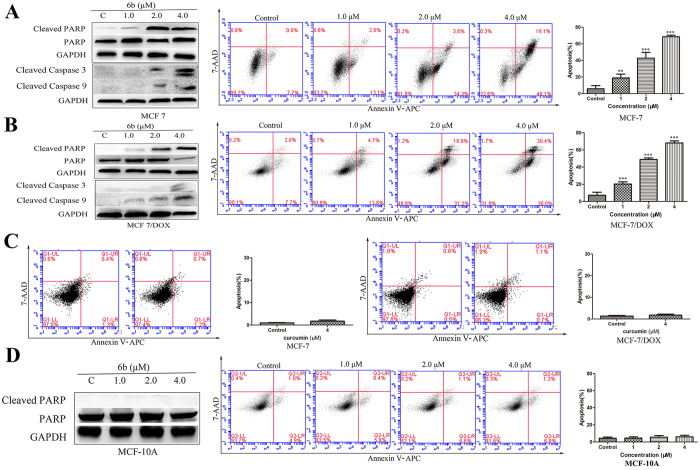
Compound 6b induced MCF-7, MCF-7/DOX but MCF-10A cells apoptosis *in Vitro* with curcumin as reference. Western blot analysis of Cleaved-PARP, Cleaved Caspase-3 and Cleaved Caspase-9 levels in whole-cell lysates. Annexin V-APC/7-AAD staining was carried out and the percentages of apoptotic cells were further determined using flow cytometry MCF-7 (**A**), MCF-7/DOX (**B**) and MCF-10A (D) cells were incubated with **6b** at different concentrations for 24 h with curcumin (**C**) as reference. **p < 0.01, ***p < 0.001.

How **6b** regulated the apoptosis-related STAT3 target genes, Bcl-2 and Bax, has been elucidated. Next, we investigated the mechanism of cancer cell apoptosis induced by **6b**. Compound **6b** induced the cleavage of PARP, Caspase 9 and Caspase 3 in MCF-7 and MCF-7/DOX cells as detected by western blot assays. We further investigated the effects of compound **6b** on inducing tumor cell apoptosis by flow cytometry. The results showed that **6b** induced the apoptosis of MCF-7 and MCF-7/DOX cells in a dose-dependent manner. The increases in apoptosis rates at 0, 1, 2, and 4 μM **6b** were 8.5%, 15.7%, 37.9% and 67.2%, respectively, in MCF-7 cells, and 9.7%, 18.5%, 50.1% and 66.4%, respectively, in MCF-7/DOX cells (Fig. 6A and B) compared to the small effect of the mother compound curcumin at 4 μM on cell apoptosis in MCF-7 and MCF-7/DOX cells (Fig. 6C). Similar to data from the MTT assay, there was little cleaved PARP and apoptosis induced by treatment with **6b** at concentrations up to 4 μM in MCF-10A cells (Fig. 6D), suggesting that **6b** is a selective antitumor agent.

**Figure 7:**
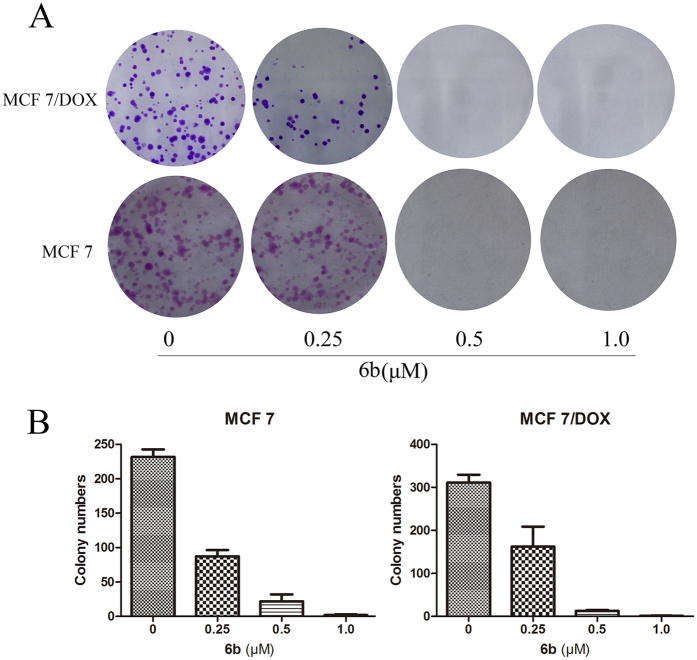
Compound 6b inhibited the colony formation of MCF-7 and MCF-7/DOX carcinoma cells. MCF-7 and MCF-7/DOX cells were treated with compound **6b** at 0.25–1 μM for 24 h and cultured for 14 d until the colonies were visible. Crystal violet solution was used to stain the colonies for 4 h and imaged (A) and counted (B).

To further investigate the anti-proliferative activity of **6b** on cancer cells, a colony survival assay was performed. As shown in Fig. 7, there was a significant reduction in clonogenic ability with 0.25 μM of compound **6b** and almost a cessation of colony formation at 0.5 μM of compound **6b**.

### Compound 6b induced cell cycle arrest in breast cancer cells

**Figure 8:**
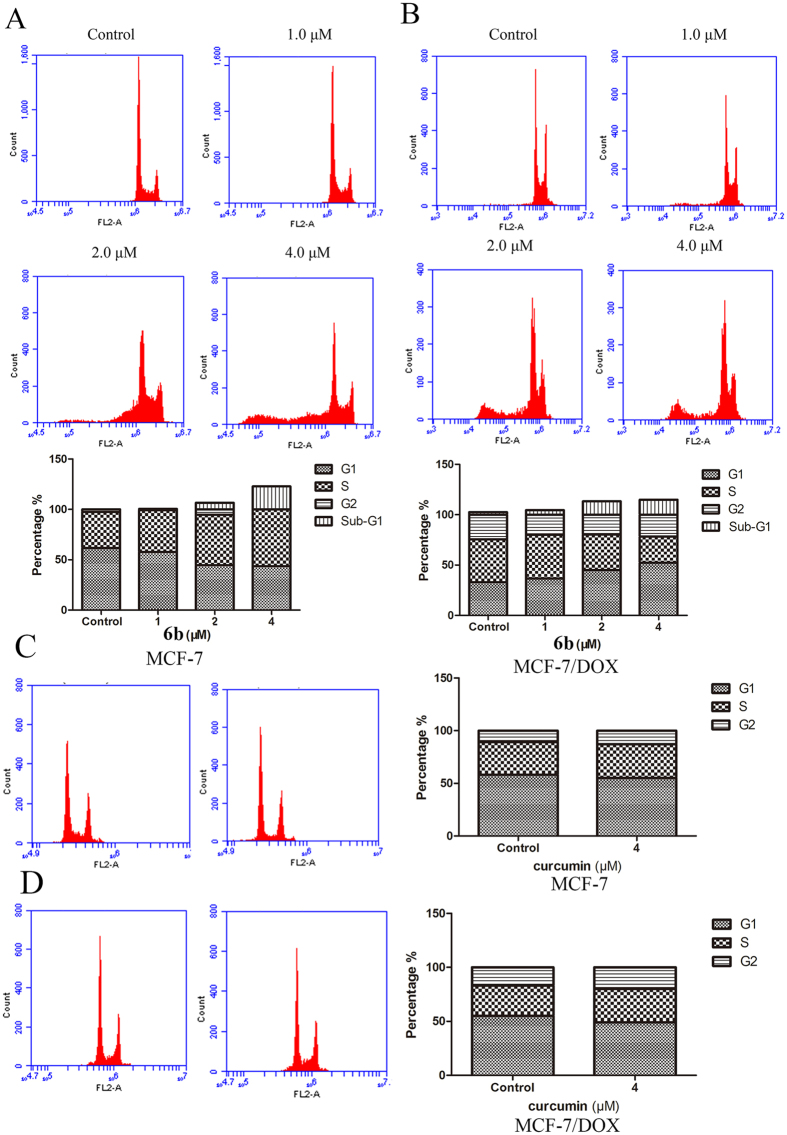
The effects of compound 6b and curcumin on cell cycle. MCF-7 and MCF-7/DOX cells were incubated with **6b** (**A** and **B**) and curcumin (**C** and **D**) at different concentrations for 24 h. Cells were harvested, washed with cold PBS, and fixed with precooled 70% ethanol incubated for a minimum of 4 h at 4 °C. The cells were then centrifuged, carefully aspirated from the supernatant, and then these cells were re-suspended in propidium iodide and RNase A solution. The suspension was incubated at 37 °C for 30 min prior to analysis by flow cytometry.

We investigated the effect of **6b** on the cell cycle in breast cancer cells by flow cytometry. As shown in Fig. 8A and B, there was a significant increase in cells in the Sub-G1 phase after treatment of MCF-7 and MCF-7/DOX cells with **6b** which further suggested that **6b** induced cancer cell apoptosis. In MCF-7/DOX cells, **6b** also increased the percentage of cells in the G0/G1 phase, which was related to a decrease in Cyclin D1. In MCF-7 cells treated with **6b**, the percentage of cells in S phase increased as well. Treatment with the mother compound, curcumin at 4 μM had little effect on the cycles of MCF-7 and MCF-7/DOX cells (Fig. 8C). These data suggest that **6b** could induce cancer cell apoptosis and cell cycle arrest.

### Compound 6b induced the generation of ROS in breast cancer cells

**Figure 9:**
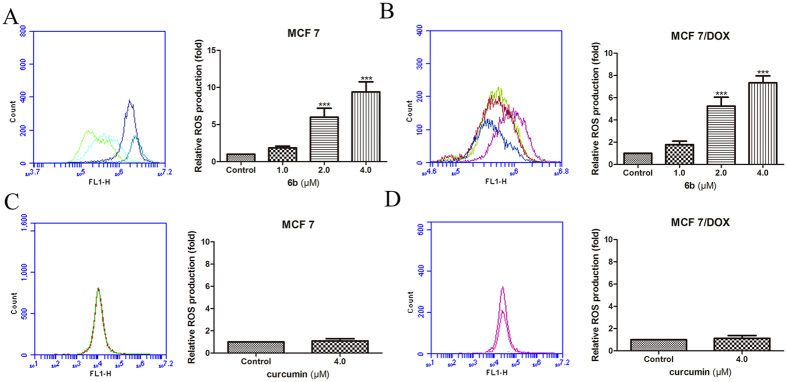
Compound 6b induced more ROS generation than curcumin. MCF-7 and MCF-7/DOX cells were incubated with **6b** (**A** and **B**) and curcumin (**C** and **D**) at different concentrations for 24 h, and then cells were collected and stained by DCFH-DA and subjected to flow cytometry. ***p < 0.001, **p < 0.01.

Curcumin and BTP derivatives induce the generation of reactive oxygen species, which would partially contribute to cell apoptosis^20^,^37^. We evaluated the effect of **6b** on the ROS levels in tumor cells using flow cytometry. As expected, the ROS levels increased 2–8-fold at different concentrations of **6b** in MCF-7 and MCF-7/DOX cells (Fig. 9A and B) while there was little effect of treatment with the mother compound curcumin at 4 μM on ROS generation in MCF-7 and MCF-7/DOX cells (Fig. 9C).

### Compound 6b significantly inhibited STAT3 phosphorylation induced by interleukin-6 (IL-6) over IFN-γ induced p-STAT1 without affecting the related kinases

**Figure 10:**
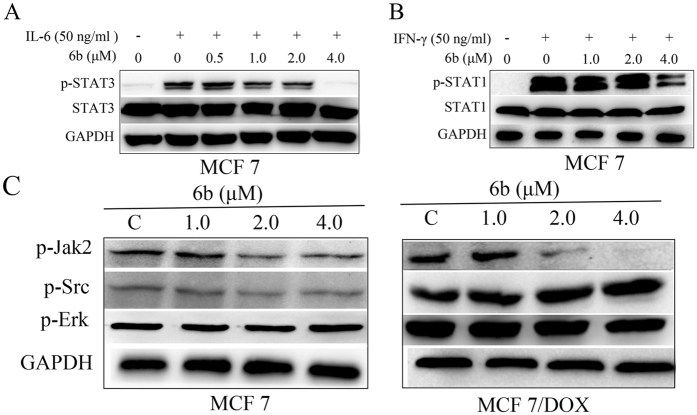
Compound 6b significantly inhibited STAT3 phosphorylation induced by interleukin-6 (IL-6) over IFN-γ induced p-STAT1 without affecting the related kinases. MCF-7 cells were seeded in 6-well plates and allowed to adhere overnight. The following day, the cells were serum-starved. The cells were then left untreated or were treated with 6b (0.5–4 μM). After 6 h, the untreated and 6b treated cells were stimulated by IL-6 (50 ng/mL) (**A**) or IFN-γ (50ng/mL) (**B**). The cells were harvested after 30 min and analyzed by western blot. (**C**) Western blot analysis of p-Jak2, p-Src and p-Erk levels in whole-cell lysates of equal total protein prepared from MCF-7 and MCF-7/DOX cells treated with compound 6b for 24 h.

The effect of **6b** on IL-6-induced STAT3 phosphorylation and IFN-γ-induced STAT1 phosphorylation was also assessed. As shown in Fig. 10A, IL-6 significantly increased the phosphorylation levels of STAT3, which was decreased by **6b** at low concentration. Treatment with 4 μM **6b** almost completely abolished STAT3 phosphorylation induced by IL-6. Treatment with **6b** had less of an effect on IFN-γ-induced STAT1 phosphorylation compared with its effect on IL-6-induced STAT3 phosphorylation (Fig. 10B). These data show that compound **6b** is a selective STAT3 inhibitor.

The activation of STAT3 phosphorylated at Tyr705 residue may be mediated by upstream kinases (such as Jak2 and Src). To further investigate the selective targeting of STAT3 by **6b**, we evaluated the effect of **6b** on p-Jak2, p-Src, p-Erk1/2 protein levels by western blot assays. Compound **6b** had little effect on p-Src and p-Erk1/2 expression but inhibited the phosphorylation of Jak2 (Fig. 10C). Further analysis showed that the p-STAT3 level was significantly inhibited by 1.0 μM **6b** in MCF-7 and MCF-7/DOX cells and that the inhibition occurred prior to the decrease of p-Jak2, indicating that inhibition of STAT3 was a Jak2-independent event. The data suggest that **6b** mainly, or directly, suppresses p-STAT3 expression, independent of these related kinases.

### Compound 6b exhibited anticancer activity *in vivo*

**Figure 11:**
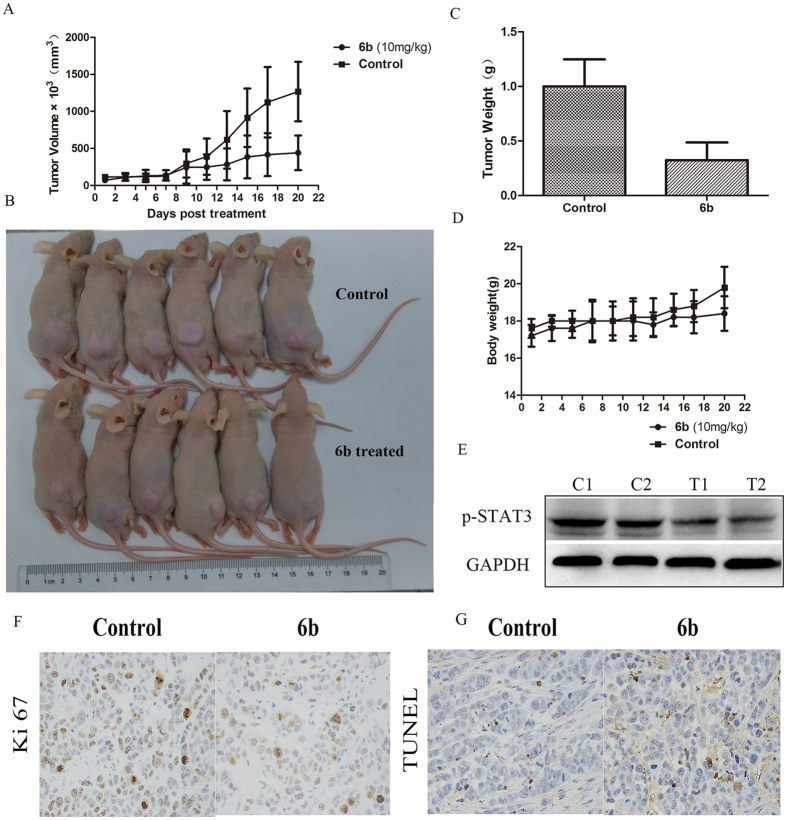
Compound 6b inhibited the growth of mouse sarcoma xenograft tumor *in vivo*. (**A**) The growing curves of mice’s tumor size. (**B**) The treated and control mice before sacrificed. (**C**) The weight of tumor tissues of treated and control mice. (**D**) The growing curves of mice’s body weight. (**E**) Western blot analysis of p-STAT3 and STAT3 levels in tumor tissues prepared from control (C1, C2) or mice treated compound **6b** (T1, T2). (**F**) Immunohistochemical staining for Ki-67 in tumour tissues obtained from the control and **6b**-treated mice. (**F**) Apoptosis of tumor cells in mice treated with **6b**, as determined by TUNEL assay.

To evaluate the *in vivo* anticancer activity of **6b**, BALB/c nude mice were inoculated subcutaneously with human MCF-7 breast cancer cells. After the establishment of solid tumors, the mice were randomly treated by intraperitoneal injection with the indicated doses of **6b** or the vehicle (Cremophor-EL/DMSO/PBS (1:1:8)) daily for 21 consecutive days. Treatment with 10 mg/kg of **6b** significantly reduced the volumes of the implanted human breast tumors (Fig. 11A and B). Importantly, the weight of the tumor tissues from mice treated with **6b** was significantly reduced by 68.2% compared with the control (Fig. 11C), and there were no significant changes in the body weights of the mice (Fig. 11D). Immunoblotting analysis of lysates from the tumor tissues treated with **6b** showed suppression of p-STAT3 compared with the control tumors (Fig. 11E). Immunohistochemical staining analysis for Ki-67 in tumor tissues obtained from the control and **6b**-treated mice showed that 6b suppressed tumor cell proliferation (Fig. 11F). (Fig. 11G) Apoptosis analysis by the terminal deoxynucleotidyl transferase dUTP nick-end labeling (TUNEL) assay of tumor cells in mice treated with or without **6b** showed that **6b** induced tumor cell apoptosis. These results show that **6b** has potent antitumor activity against the growth of implanted human breast tumors (MCF-7) with little toxicity.

## Conclusions

The poor prognosis and the rapid emergence of drug resistance limits the benefits of breast cancer treatment. Over-activated STAT3 has been found in a large number of drug-sensitive and drug-resistant breast cancers, and STAT3 inhibition may be a viable treatment option for both drug-sensitive and drug-resistant breast cancers by regulating STAT3 target genes and inhibiting STAT3-mediated MDR1 gene expression^2^. Cancer cells are usually exposed to a moderate level of reactive oxygen species (ROS), primarily due to their active metabolism in response to oncogenic signals^23^. In fact, cancer cells take advantage of this moderate oxidative stress for several important processes such as proliferation, angiogenesis, and metastasis^24^. However, high levels of ROS irreversibly damage DNA and lipids and ultimately cause cancer cell apoptosis. While an exogenous ROS insult is tolerable to normal cells, it may exceed the threshold cancer cells can endure and lead to selective cytotoxicity against cancer cells^27^. In fact, there have been increasing efforts to increase the levels of ROS for what is termed “oxidation therapy,” specifically in cancer cells. A combination of STAT3 inhibition and a promotion of ROS activity may be a new strategy for the treatment of solid tumors^24^,^28^. Therefore, a new series of curcumin-BTP hybrids (**6a**-**v**) were designed, synthesized, and biologically evaluated as STAT3 inhibitors. According to our screening results, we found that **6b** exerted potent and selective antitumor activity against MCF-7 and MCF-7/DOX cells with a weak effect on MCF-10A breast epithelial cells. This is because the p-STAT3 levels in MCF-7 and MCF-7/DOX cells are significantly higher than that in MCF-10A breast epithelial cells. Docking studies revealed that **6b** could tightly bind to the SH2 domain of STAT3 suggesting that **6b** may be a STAT3-SH2 domain inhibitor. Further biological evaluation showed that **6b** inhibited persistent and IL-6-induced STAT3 phosphorylation, nuclear translocation, and DNA binding activity and regulated the expression of the STAT3 downstream genes, Bcl-2, Bax and Cyclin D1, with little effect on p-Src or p-Erk. Compound **6b** also inhibited STAT3-mediated P-gp expression in MCF-7/DOX cells. Additionally, **6b** promoted intracellular ROS production and accumulation, induced cancer cell cycle arrest and apoptosis, and abolished the colony formation activity of cancer cells. Furthermore, **6b** significantly inhibited human implanted breast cancer *in vivo* without obvious toxicity. Together, our findings highlight that hybrid compounds similar to **6b** that are novel, direct STAT3 inhibitors with ROS production activity are worth further investigation as potential therapeutics for breast cancer or drug-resistant breast cancer.

## Materials and Methods

### Chemistry

All reagent grade chemicals used were purchased from Sinopharm Chemical Reagent Co., Ltd. (China). Reaction progress was monitored using analytical thin layer chromatography (TLC) on precoated silica gel GF254 plates (Qingdao Haiyang Chemical Plant, Qingdao, China), and the spots were detected under UV light (254 nm). Melting points were measured using an XT-4 micro-melting point instrument and were uncorrected. IR (KBr-disc) spectra were recorded by a Bruker Tensor 27 spectrometer. ^1^H NMR and ^13^C NMR spectra were measured on a Bruker ACF-500 spectrometer at 25 °C and referenced to TMS. Chemical shifts were reported in ppm (*δ*) using the residual solvent line as an internal standard. Splitting patterns were designed as s, singlet; d, doublet; t, triplet; m, multiplet. Mass spectra were obtained on an MS Agilent 1100 Series LC/MSD Trap mass spectrometer (ESI-MS) and a Mariner ESI-TOF spectrometer (HRESI-MS). Column chromatography was performed on silica gel (90–150 μm; Qingdao Marine Chemical Inc). Intermediates **2a** and **2b** were prepared as previously described^38^.

#### 5-bromobenzo[b]thiophene-2-carboxylic acid (**2a**)

Yield 96%, white solid; ^1^H NMR (500 MHz, DMSO) *δ*13.60 (s, 1H, COOH), 8.17 (s, 1H), 7.95 (d, *J* = 8.6 Hz, 1H), 7.85 (s, 1H), 7.56 (d, *J* = 8.6 Hz, 1H).

#### 6-bromobenzo[b]thiophene-2-carboxylic acid (**2b**)

Yield 96%, white solid; ^1^H NMR (500 MHz, DMSO) *δ* 13.57 (s, 1H, COOH), 8.36 (s, 1H, Ar-H), 8.10 (s, 1H, Ar-H), 7.95 (d, *J* = 8.6 Hz, 1H, Ar-H), 7.61 (dd, *J* = 8.6, 1.6 Hz, 1H, Ar-H).

#### General procedures for the preparation of **3a** and **3b**

To a solution of **2a** (1.0 g, 3.9 mmol) or **2b** (1.0 g, 3.9 mmol) and HATU (1.78 g, 4.7 mmol) in the presence of trimethylamine (0.97 ml, 5.8 mmol), tert-butyl-1-piperazinecarbo-xylate (0.8 g, 4.3 mmol) was added and stirred overnight. After completion, dilute hydrochloric acid solution was added and extracted by dichloromethane, washed by saturated sodium carbonate solution three times. The combined organic layers were washed with brine and dried with sodium sulfate, filtered, and concentrated, loaded on silica gel, and purified by silica gel chromatography with petroleum/ethyl acetate as eluent to give pure **3a** or **3b**.

*Tert*-*butyl*-*4*-(*5*-*bromobenzo*[*b*]*thiophene*-*2*-*carbonyl*) *piperazine*-*1*-*carboxylat (**3a***) ^1^H NMR (500 MHz, CDCl_3_) *δ* 7.82 (d, *J* = 1.0 Hz, 1H), 7.73 (d, *J* = 8.0 Hz, 1H), 7.68 (dd, *J* = 8.0, 1.5 Hz, 1H), 5.72–4.47 (m, 8 H) 1.46 (s, 9H).

*Tert*-*butyl*-*4*-(*5*-*bromobenzo*[*b*]*thiophene*-*2*-*carbonyl*) *piperazine*-*1*-*carboxylat (**3b***) ^1^H NMR (500 MHz, CDCl_3_) *δ* 8.00 (d, *J* = 4.7 Hz, 1H), 7.67 (d, *J* = 8.5 Hz, 1H), 7.53–7.48 (m, 1H), 7.44 (s, 1H), 3.78–3.68 (m, 4H), 3.58–3.46 (m, 4H), 1.48 (s, 9H).

#### General procedures for the preparation of **4a** and **4b**

3-Chloroperbenzoic acid (1.27 g, 5.9 mmol, 80%) was added, in portions, to the stirred solution of compound **3a** or **3b** (1.0 g, 2.35 mmol) in dichloromethane (50 ml) over a 20–30 minute period. The mixture was heated to reflux for approximately 5 h and monitored by TLC. After completion, the mixture was cooled to room temperature and sodium hydrogen sulfite (20 ml) was added to it. The solution was stirred for 15 minutes and extracted with dichloromethane. The organic phase was washed with aqueous sodium bicarbonate. The organic phase was separated, washed with brine and dried with sodium sulfate, filtered, and the solvents were removed under reduced pressure, loaded onto a silica gel, and purified by silica gel chromatography with petroleum/ethyl acetate as an eluent to give pure ***4a*** or ***4b***.

*Tert*-*butyl*-*4*-(*5*-*bromo*-*1*,*1*-*dioxidobenzo*[*b*]*thiophene*-*2*-*carbonyl*)*piperazine*-*1*-*carboxylate (**4a***) ^1^H NMR (500 MHz, CDCl_3_) *δ* 7.89 (d, *J* = 1.0 Hz, 1H), 7.82 (d, *J* = 8.0 Hz, 1H), 7.77 (dd, *J* = 8.0, 1.5 Hz, 1H), 5.72 (t, *J* = 5.9 Hz, 4H), 4.47 (dd, *J* = 5.8, 1.7 Hz, 4H) 1.46 (s, 9H).

*Tert*-*butyl*-*4*-(*6*-*bromo*-*1*,*1*-*dioxidobenzo*[*b*]*thiophene*-*2*-*carbonyl*)*piperazine*-*1*-*carboxylate (**4b***) ^1^H NMR (500 MHz, CDCl_3_) *δ* 7.86 (s, 1H), 7.76–7.71 (m, 1H), 7.32–7.27 (m, 2H), 3.55–3.50 (m, 8H), 1.47 (s, 9H).

#### General procedures for the preparation of **5a** and **5b**

A solution of **4a** or **4b** in chlorine hydride gas in methanol and dichloromethane (1:1) (10 ml) was stirred for 48 h, after completion determined by TLC, the solvents were removed under reduced pressure to generate compound **5a** or **5b**.

(*5*-*bromo*-*1*,*1*-*dioxidobenzo*[*b*]*thiophen*-*2*-*yl*)(*piperazin*-*1*-*yl*)*methanone hydrochloride (**5a***) ^1^H NMR (500 MHz, DMSO) *δ* 7.98 (s, 1H), 7.92 (s, 2H), 7.84 (s, 1H), 3.83 (s, 4H), 3.16 (d, *J* = 3.6 Hz, 4H).

(*5*-*bromo*-*1*,*1*-*dioxidobenzo*[*b*]*thiophen*-*2*-*yl*)(*piperazin*-*1*-*yl*)*methanone hydrochloride (**5b***) ^1^H NMR (500 MHz, DMSO) *δ* 7.90 (s, 1H), 7.86–7.80 (m, 1H), 7.39–7.32 (m, 2H), 3.65–3.59 (m, 8H).

#### General procedures for the preparation of **6a**-**v**

To a solution of **5a** (0.1 g, 0.25 mmol) or **5b** (0.1 g, 0.25 mmol) and HATU (0.096 g, 0.27 mmol) in the presence of trimethylamine (0.07 ml, 0.508 mmol), a different cinnamic acid (0.28 mmol) was added and the solution was stirred overnight. After completion, dilute hydrochloric acid solution was added and the resulting product was extracted with dichloromethane and washed with a saturated sodium carbonate solution three times. The combined organic layers were washed with brine and dried sodium sulfate, were filtered and concentrated, loaded onto a silica gel, and purified by silica gel chromatography with dichloromethane/ethyl acetate as an eluent to give the pure compounds **6a**-**v**.

(*E*)-*1*-(*4*-(*5*-*bromo*-*1*,*1*-*dioxidobenzo*[*b*]*thiophene*-*2*-*carbonyl*)*piperazin*-*1*-*yl*)-*3*-(*3*,*4*-*dimethoxyphenyl*)*prop*-*2*-*en*-*1*-*one (**6a***) Yield 94%, white solid; m.p. 244–246 °C; 3450, 2921, 1642, 1592, 1517, 1441, 1309, 1257, 1144, 1018, 993, 909, 810, 726, 616, 550 cm^−1^; ^1^H NMR (500 MHz, DMSO) *δ* 7.96 (s, 1H), 7.92 (s, 2H), 7.78 (s, 1H), 7.48 (d, *J* = 15.2 Hz, 1H), 7.36 (s, 1H), 7.22 (d, *J* = 8.0 Hz, 1H), 7.14 (d, *J* = 14.8 Hz, 1H), 6.97 (d, *J* = 8.3 Hz, 1H), 3.86–3.76 (m, 8H), 3.64 (s, 6H). ^13^C NMR (126 MHz, DMSO) *δ* 165.51 (s), 158.22 (s), 150.93 (s), 149.48 (s), 142.77 (s), 137.80 (s), 137.78 (s), 132.02 (s), 129.20 (s), 128.96 (s), 128.41 (s), 125.47 (s), 125.09 (s), 122.91 (s), 115.83 (s), 112.14 (s), 111.07 (s), 56.16 (s), 56.24 (s). MS (ESI) *m*/*z* 547.0 [M + H]^+^; HRMS (ESI) *m*/*z* 547.0532 [M + H]^+^ (calcd for 547.0533, C_24_H_24_BrN_2_O_6_S).

(*E*)-*1*-(*4*-(*5*-*bromo*-*1*,*1*-*dioxidobenzo*[*b*]*thiophene*-*2*-*carbonyl*)*piperazin*-*1*-*yl*)-*3*-(*4*-*methoxyphenyl*)*prop*-*2*-*en*-*1*-*one (**6b***) Yield 92%, white solid; m.p. 250–251 °C; 3448, 1644, 1603, 1512, 1431, 1314, 1225, 1176, 1156, 1064, 997, 967, 821, 745, 570 cm^−1^; ^1^H NMR (500 MHz, DMSO) *δ* 7.98 (s, 1H), 7.97–7.91 (m, 2H), 7.80 (s, 1H), 7.70 (d, *J* = 8.1 Hz, 2H), 7.51 (d, *J* = 15.3 Hz, 1H), 7.23–7.06 (m, 1H), 6.99 (d, *J* = 8.6 Hz, 2H), 3.81 (s, 5H), 3.66 (s, 6H). ^13^C NMR (126 MHz, DMSO) *δ* 158.94 (s), 151.92 (s), 136.82 (s), 130.02 (s), 129.69 (s), 127.16 (s), 126.46 (s), 125.49, 125.34, 123.43 (s), 119.12 (s), 111.66 (s), 110.60 (s), 54.47 (s). MS (ESI) *m*/*z* 517.0 [M + H]^+^; HRMS (ESI) *m*/*z* 517.0426 [M + H]^+^ (calcd for 517.0427, C_23_H_22_BrN_2_O_5_S).

(*E*)-*1*-(*4*-(*5*-*bromo*-*1*,*1*-*dioxidobenzo*[*b*]*thiophene*-*2*-*carbonyl*)*piperazin*-*1*-*yl*)-*3*-(*3*,*4*,*5*-*trimethoxyphenyl*)*prop*-*2*-*en*-*1*-*one (**6c***) Yield 88%, white solid; m.p. 94–96 °C; 3448, 2936, 1643, 1505, 1426, 1312, 1266, 1154, 1124, 1062, 992, 823, 723, 577 cm^−1^; ^1^H NMR (500 MHz, DMSO) *δ* 7.98 (s, 1H), 7.97–7.90 (m, 2H), 7.81 (s, 1H), 7.50 (d, *J* = 15.3 Hz, 1H), 7.29–7.18 (m, 1H), 7.06 (d, *J* = 13.0 Hz, 2H), 3.94–3.78 (m, 8H), 3.78–3.58 (m, 9H). ^13^C NMR (126 MHz, DMSO) *δ* 164.75 (s), 162.21 (s), 157.54 (s), 152.99 (s), 142.25 (s), 138.97 (s), 134.54 (s), 134.20 (s), 131.53 (s), 130.81 (s), 130.53 (s), 129.78 (s), 127.58 (s), 123.09 (s), 116.94 (s), 105.76 (s), 60.02 (s), 56.05 (s), 38.16 (s), 35.68 (s). MS (ESI) *m*/*z* 577.0 [M + H]^+^; HRMS (ESI) *m*/*z* 577.0635 [M + H]^+^ (calcd for 577.0635, C_25_H_26_BrN_2_O_7_S).

(*E*)-*1*-(*4*-(*5*-*bromo*-*1*,*1*-*dioxidobenzo*[*b*]*thiophene*-*2*-*carbonyl*)*piperazin*-*1*-*yl*)-*3*-(*4*-*hydroxy*-*3*,*5*-*dimethoxyphenyl*)*prop*-*2*-*en*-*1*-*one (**6d***) Yield 76%, white solid; m.p. 125–127 °C; 3433, 1641, 1514, 1428, 1311, 1219, 1155, 1113, 993, 842, 724, 579 cm^−1^; ^1^H NMR (500 MHz, DMSO) *δ* 8.81 (s, 1H), 7.96 (s, 1H), 7.92 (s, 2H), 7.78 (s, 1H), 7.46 (d, *J* = 15.2 Hz, 1H), 7.16–7.04 (m, 1H), 7.02 (s, 2H), 3.81–3.65 (m, 14H). ^13^C NMR (126 MHz, DMSO) *δ* 165.19 (s), 157.66 (s), 148.09(s), 143.07 (s), 137.73 (s), 134.61 (s), 134.33 (s), 131.60 (s), 130.94 (s), 129.89 (s), 127.72 (s), 125.47 (s), 123.18 (s), 114.64 (s), 106.17 (s), 56.23 (s), 30.67 (s). MS (ESI) *m*/*z* 563.0 [M + H]^+^; HRMS (ESI) *m*/*z* 563.0484 [M + H]^+^ (calcd for 563.0482, C_24_H_24_BrN_2_O_7_S).

(*E*)-*1*-(*4*-(*5*-*bromo*-*1*,*1*-*dioxidobenzo*[*b*]*thiophene*-*2*-*carbonyl*)*piperazin*-*1*-*yl*)-*3*-(*4*-*nitrophenyl*)*prop*-*2*-*en*-*1*-*one (**6e***) Yield 86%, white solid; m.p. >250 °C; 3445, 1634, 1611, 1562, 1520, 1432, 1345, 1310, 1264, 1196, 1157, 1067, 992, 927, 841, 753, 576 cm^−1^; ^1^H NMR (500 MHz, DMSO) *δ* 8.25 (d, *J* = 8.6 Hz, 2H), 8.01 (d, *J* = 8.6 Hz, 2H), 7.96 (s, 1H), 7.94–7.88 (m, 2H), 7.78 (s, 1H), 7.62 (d, *J* = 15.4 Hz, 1H), 7.52 (d, *J* = 15.4 Hz, 1H), 3.86–3.75 (m, 8H). ^13^C NMR (126 MHz, DMSO) δ 165.13 (s), 157.62 (s), 148.06 (s), 143.03 (s), 137.71 (s), 134.59 (s), 134.29 (s), 131.58 (s), 130.90 (s), 129.86 (s), 127.68 (s), 125.44 (s), 123.16 (s), 114.61 (s), 106.16 (s), 56.20 (s), 30.64 (s). MS (ESI) *m*/*z* 532.0 [M + H]^+^; HRMS (ESI) *m*/*z* 532.0173[M + H]^+^ (calcd for 532.0172, C_22_H_19_BrN_3_O_6_S).

(*E*)-*1*-(*4*-(*5*-*bromo*-*1*,*1*-*dioxidobenzo*[*b*]*thiophene*-*2*-*carbonyl*)*piperazin*-*1*-*yl*)-*3*-(*pyridin*-*2*-*yl*)*prop*-*2*-*en*-*1*-*one (**6f***) Yield 65%, white solid; m.p. 241–242 °C; 3449, 3085, 2919, 1651, 1608, 1565, 1427, 1316, 1275, 1226, 1204, 1157, 1065, 994, 970, 938, 886, 817, 779, 746, 592, 569, 553, 453, 426 cm^−1^; ^1^H NMR (500 MHz, DMSO) *δ* 8.62 (d, *J* = 3.9 Hz, 1H), 7.96 (s, 1H), 7.94–7.89 (m, 2H), 7.86–7.83 (m, 1H), 7.78 (s, 1H), 7.74 (d, *J* = 7.6 Hz, 1H), 7.59–7.51 (m, 2H), 7.41–7.33 (m, 1H), 3.76–3.66 (m, 8H). ^13^C NMR (126 MHz, DMSO) *δ* 164.36 (s), 157.56 (s), 153.09 (s), 149.68 (s), 141.09 (s), 136.99 (s), 134.55 (s), 134.20 (s), 131.53 (s), 130.81 (s), 129.80 (s), 127.58 (s), 124.04 (s), 123.85 (s), 123.10 (s), 121.45 (s). MS (ESI) *m*/*z* 488.0 [M + H]^+^; HRMS (ESI) *m*/*z* 488.0275 [M + H]^+^ (calcd for 488.0274, C_21_H_19_BrN_3_O_4_S).

(*E*)-*1*-(*4*-(*5*-*bromo*-*1*,*1*-*dioxidobenzo*[*b*]*thiophene*-*2*-*carbonyl*)*piperazin*-*1*-*yl*)-*3*-(*4*-*bromophenyl*)*prop*-*2*-*en*-*1*-*one (**6g***) Yield 97%, white solid; m.p. >250 °C; 3450, 3079, 2916, 1644, 1601, 1564, 1486, 1429, 1314, 1266, 1226, 1205, 1156, 1067, 1044, 998, 967, 879, 813, 744, 589, 568, 552, 486, 452, 426 cm^−1^; ^1^H NMR (500 MHz, DMSO) *δ* 7.96 (s, 1H), 7.93 (d, *J* = 8.4 Hz, 2H), 7.78 (s, 1H), 7.70 (d, *J* = 7.3 Hz, 2H), 7.61 (d, *J* = 8.4 Hz, 2H), 7.49 (d, *J* = 15.4 Hz, 1H), 7.39–7.27 (m, 1H), 3.86–3.76 (m, 8H). ^13^C NMR (126 MHz, DMSO) *δ* 164.45 (s), 157.54 (s), 140.44 (s),136.48 (s), 134.53 (s), 134.31 (s), 132.25 (s), 131.61 (s), 131.25 (s), 131.42 (s), 130.83 (s), 129.92 (s), 129.78 (s), 127.87 (s), 127.57 (s), 125.30 (s), 123.09 (s), 122.74 (s), 118.94 (s). MS (ESI) *m*/*z* 566.9 [M + H]^+^; HRMS (ESI) *m*/*z* 566.9404 [M + H]^+^ (calcd for 566.9407, C_22_H_19_Br_2_N_2_O_4_S).

(*E*)-*1*-(*4*-(*5*-*bromo*-*1*,*1*-*dioxidobenzo*[*b*]*thiophene*-*2*-*carbonyl*)*piperazin*-*1*-*yl*)-*3*-(*4*-*chlorophenyl*)*prop*-*2*-*en*-*1*-*one (**6h***) Yield 95%, white solid; m.p. 225–227 °C; 3080, 2917, 1645, 1491, 1429, 1314, 1266, 1226, 1204, 1156, 1065, 998, 968, 880, 817, 744, 590, 569, 552, 490, 455, 423 cm^−1^; ^1^H NMR (500 MHz, DMSO) *δ* 7.96 (s, 1H), 7.94–7.88 (m, 2H), 7.77 (m, 3H), 7.49 (m, 3H), 7.32 (d, *J* = 14.9 Hz, 1H), 3.86–3.72 (m, 8H). ^13^C NMR (126 MHz, DMSO) *δ* 164.45 (s), 157.54 (s), 140.36 (s), 134.53 (s), 134.19 (s), 133.97 (s), 131.52 (s), 130.83 (s), 129.78 (s), 129.68 (s), 128.68 (s), 127.58 (s), 123.09 (s), 118.88 (s), 30.64 (s). MS (ESI) *m*/*z* 523.0 [M + H]^+^; HRMS (ESI) *m*/*z* 522.9009 [M + H]^+^ (calcd for 522.9911, C_22_H_19_BrClN_2_O_4_S).

(*E*)-*1*-(*4*-(*5*-*bromo*-*1*,*1*-*dioxidobenzo*[*b*]*thiophene*-*2*-*carbonyl*)*piperazin*-*1*-*yl*)-*3*-(*4*-(*trifluoromethyl*)*phenyl*)*prop*-*2*-*en*-*1*-*one (**6i***) Yield 95%, white solid; m.p. 245–247 °C; 3443, 3084, 1603, 1431, 1329, 1268, 1228, 1205, 1168, 1067, 999, 968, 954, 827, 744, 590, 568, 453, 423 cm^−1^; ^1^H NMR (500 MHz, DMSO) *δ* 8.05–7.87 (m, 5H), 7.83–7.71 (m, 3H), 7.58 (d, *J* = 15.4 Hz, 1H), 7.46 (t, *J* = 12.2 Hz, 1H), 3.82–3.66 (m, 8H). ^13^C NMR (126 MHz, DMSO) *δ* 164.25 (s), 157.57 (s), 139.93 (s), 139.07 (s), 134.54 (s), 134.20 (s), 131.54 (s), 130.87 (s), 129.79 (s), 129.40 (s), 129.14 (s), 128.63 (d, *J* = 15.8 Hz), 127.58 (s), 125.48 (d, *J* = 3.8 Hz), 125.12 (s), 123.02 (d, *J* = 18.3 Hz), 121.05 (s). MS (ESI) *m*/*z* 555.0 [M + H]^+^; HRMS (ESI) *m*/*z* 555.0191 [M + H]^+^ (calcd for 555.0196, C_23_H_19_BrF_3_N_2_O_4_S).

(*E*)-*1*-(*4*-(*5*-*bromo*-*1*,*1*-*dioxidobenzo*[*b*]*thiophene*-*2*-*carbonyl*)*piperazin*-*1*-*yl*)-*3*-(*4*-*hydroxy*-*3*-*methoxyphenyl*)*prop*-*2*-*en*-*1*-*one (**6j***) Yield 64%, white solid; m.p.127–129 °C; 3397, 2923, 1641, 1514, 1432, 1311, 1155, 1063, 993, 842, 725, 557 cm^−1^; ^1^H NMR (500 MHz, DMSO) *δ* 9.43 (s, 1H), 7.96–7.95 (m, 2H), 7.93–7.93 (m, 2H), 7.78 (s, 1H), 7.45 (d, *J* = 15.2 Hz, 1H), 7.33 (s, 1 H), 7.10 (d, *J* = 8.1 Hz, 1H), 6.78 (d, *J* = 8.1 Hz, 1H), 3.87–3.58 (m, 11H). ^13^C NMR (126 MHz, DMSO) *δ* 165.06 (s), 162.21 (s), 157.54 (s), 148.55 (s), 147.80 (s), 142.56 (s), 134.55 (s), 134.20 (s), 131.53 (s), 130.79 (s), 129.78 (s), 127.58 (s), 126.55 (s), 123.09 (s), 122.53 (s), 115.42 (s), 114.22 (s), 111.28 (s), 55.77 (s), 38.16 (s), 35.68 (s). MS (ESI) *m*/*z* 533.0 [M + H]^+^; HRMS (ESI) *m*/*z* 533.0380 [M + H]^+^ (calcd for 533.0376, C_23_H_22_BrN_2_O_6_S).

(*E*)-*1*-(*4*-(*5*-*bromo*-*1*,*1*-*dioxidobenzo*[*b*]*thiophene*-*2*-*carbonyl*)*piperazin*-*1*-*yl*)-*3*-(*4*-*fluorophenyl*)*prop*-*2*-*en*-*1*-*one (**6k***) Yield 91%, white solid; m.p. >250 °C; 3445, 3083, 1602, 1509, 1430, 1314, 1269, 1221, 1157, 1065, 997, 967, 824, 744, 729, 590, 569, 552, 512, 422 cm^−1^; ^1^H NMR (500 MHz, DMSO) *δ* 7.96 (s, 1H), 7.92 (s, 2H), 7.85–7.74 (m, 3H), 7.52 (d, *J* = 15.4 Hz, 1H), 7.25 (t, *J* = 8.8 Hz, 3H), 3.72 (d, *J* = 80.2 Hz, 8H). ^13^C NMR (126 MHz, DMSO) *δ* 164.58 (s), 161.76 (s), 157.54 (s), 140.57 (s), 134.53 (s), 134.19 (s), 131.59 (d, *J* = 16.7 Hz), 130.83 (s), 130.18 (d, *J* = 8.4 Hz), 129.78 (s), 127.58 (s), 123.09 (s), 117.91 (s), 115.70 (s), 115.53 (s). MS (ESI) *m*/*z* 505.0 [M + H]^+^; HRMS (ESI) *m*/*z* 505.0225 [M + H]^+^ (calcd for 505.0227, C_22_H_19_BrFN_2_O_4_S).

(*E*)-*1*-(*4*-(*6*-*bromo*-*1*,*1*-*dioxidobenzo*[*b*]*thiophene*-*2*-*carbonyl*)*piperazin*-*1*-*yl*)-*3*-(*3*,*4*-*dimethoxyphenyl*)*prop*-*2*-*en*-*1*-*one (**6l***) Yield 87%, white solid; m.p. 222–224 °C; 3422, 1641, 1600, 1511, 1438, 1308, 1260, 1218, 1156, 1073, 1030, 993, 967, 806, 607, 548 cm^−1^; ^1^H NMR (500 MHz, DMSO) *δ* 8.32 (s, 1H), 8.00 (dd, *J* = 8.0, 1.6 Hz, 1H), 7.87 (s, 1H), 7.66 (d, *J* = 8.0 Hz, 1H), 7.50 (d, *J* = 15.3 Hz, 1H), 7.38 (s, 1H), 7.24 (d, *J* = 8.1 Hz, 1H), 7.15 (d, *J* = 15.2 Hz, 1H), 6.99 (d, *J* = 8.3 Hz, 1H), 3.91–3.76 (m, 8 H), 3.68 (s, 6H). ^13^C NMR (126 MHz, DMSO) *δ* 164.93 (s), 157.64 (s), 150.36 (s), 148.91 (s), 142.20 (s), 137.23 (d, *J* = 5.6 Hz), 131.45 (s), 128.63 (s), 128.39 (s), 127.84 (s), 124.90 (s), 124.51 (s), 122.34 (s), 115.26 (s), 111.57 (s), 110.50 (s), 55.59 (d, *J* = 19.4 Hz). MS (ESI) *m*/*z* 547.0 [M + H]^+^; HRMS (ESI) *m*/*z* 547.0531 [M + H]^+^ (calcd for 547.0533, C_24_H_24_BrN_2_O_6_S).

(*E*)-*1*-(*4*-(*6*-*bromo*-*1*,*1*-*dioxidobenzo*[*b*]*thiophene*-*2*-*carbonyl*)*piperazin*-*1*-*yl*)-*3*-(*4*-*methoxyphenyl*)*prop*-*2*-*en*-*1*-*one (**6m***) Yield 89%, white solid; m.p. 220–222 °C; 3422, 1644, 1621, 1511, 1427, 1311, 1226, 1176, 1153, 1073, 1028, 996, 817, 745, 589, 545 cm^−1^; ^1^H NMR (500 MHz, DMSO) *δ* 8.33 (s, 1H), 8.00 (dd, *J* = 8.0, 1.5 Hz, 1H), 7.86 (s, 1H), 7.68 (dd, *J* = 14.3, 8.3 Hz, 3H), 7.51 (d, *J* = 15.3 Hz, 1H), 7.20–7.09 (m, 1H), 6.99 (d, *J* = 8.6 Hz, 2H), 3.82 (s, 5H), 3.67 (s, 6H). ^13^C NMR (126 MHz, DMSO) *δ* 164.91 (s), 160.45 (s), 157.65 (s), 141.68 (s), 137.20 (s), 131.46 (s), 129.63 (s), 128.64 (s), 128.39 (s), 127.63 (s), 124.90 (s), 124.52 (s), 115.25 (s), 114.15 (s), 55.21 (s), 38.16 (s). MS (ESI) *m*/*z* 517.0 [M + H]^+^; HRMS (ESI) *m*/*z* 517.0430 [M + H]^+^ (calcd for 517.0427, C_23_H_22_BrN_2_O_5_S).

(*E*)-*1*-(*4*-(*6*-*bromo*-*1*,*1*-*dioxidobenzo*[*b*]*thiophene*-*2*-*carbonyl*)*piperazin*-*1*-*yl*)-*3*-(*3*,*4*,*5*-*trimethoxyphenyl*)*prop*-*2*-*en*-*1*-*one (**6n***) Yield 76%, white solid; m.p. 81–85 °C; 3449, 2928, 1643, 1505, 1425, 1314, 1252, 1225, 992, 825, 608, 549 cm^−1^; ^1^H NMR (500 MHz, DMSO) *δ* 8.34 (s, 1H), 8.00 (dd, *J* = 8.0, 1.7 Hz, 1H), 7.87 (s, 1H), 7.67 (d, *J* = 8.1 Hz, 1H), 7.51 (d, *J* = 15.3 Hz, 1H), 7.23 (d, *J* = 14.5 Hz, 1H), 7.08 (s, 2H), 3.84 (d, *J* = 5.2 Hz, 8H), 3.69 (d, *J* = 13.6 Hz, 9H). ^13^C NMR (126 MHz, DMSO) *δ* 164.74 (s), 157.65 (s), 152.99 (s), 142.27 (s), 138.97 (s), 137.21 (s), 131.46 (s), 130.54 (s), 128.63 (s), 128.38 (s), 124.90 (s), 124.52 (s), 116.93 (s), 105.75 (s), 60.02 (s), 56.05 (s), 38.16 (s). MS (ESI) *m*/*z* 577.0 [M + H]^+^; HRMS (ESI) *m*/*z* 577.0640 [M + H]^+^ (calcd for 577.0639, C_25_H_26_BrN_2_O_7_S).

(*E*)-*1*-(*4*-(*6*-*bromo*-*1*,*1*-*dioxidobenzo*[*b*]*thiophene*-*2*-*carbonyl*)*piperazin*-*1*-*yl*)-*3*-(*4*-*hydroxy*-*3*,*5*-*dimethoxyphenyl*)*prop*-*2*-*en*-*1*-*one (**6o***) Yield 61%, white solid; m.p. 112–114 °C; 3422, 2924, 2853, 1641, 1513, 1459, 1314, 1261, 1218, 1153, 1111, 992, 822, 548 cm^−1^; ^1^H NMR (500 MHz, DMSO) δ 8.83 (s, 1H), 8.34 (s, 1H), 8.00 (dd, J = 8.0, 1.3 Hz, 1H), 7.87 (s, 1H), 7.67 (d, J = 8.1 Hz, 1H), 7.48 (d, J = 15.2 Hz, 1H), 7.12 (d, J = 15.0 Hz, 1H), 7.04 (s, 2H), 3.91–3.78 (m, 8H), 3.67 (s, 6H). ^13^C NMR (126 MHz, DMSO) δ 165.00 (s), 157.64 (s), 147.98 (s), 142.93 (s), 137.65 (s), 137.21 (s), 131.44 (s), 128.63 (s), 128.39 (s), 125.37 (s), 124.90 (s), 124.52 (s), 114.53 (s), 106.12 (s), 56.13 (s), 38.16 (s). MS (ESI) m/z 563.0 [M + H]^+^; HRMS (ESI) m/z 563.0485 [M + H]^+^ (calcd for 563.0482, C_24_H_24_BrN_2_O_7_S).

(*E*)-*1*-(*4*-(*6*-*bromo*-*1*,*1*-*dioxidobenzo*[*b*]*thiophene*-*2*-*carbonyl*)*piperazin*-*1*-*yl*)-*3*-(*2*-*hydroxy*)*prop*-*2*-*en*-*1*-*one (**6p***) Yield 56%, white solid; m.p. >250 °C; 3444, 2924, 1641, 1433, 1311, 1218, 1153, 991, 841, 758, 550 cm^−1^; ^1^H NMR (500 MHz, DMSO) *δ* 10.04 (s, 1H), 8.34 (s, 1H), 8.00 (dd, *J* = 8.0, 1.4 Hz, 1H), 7.89–7.80 (m, 2H), 7.68 (dd, *J* = 17.9, 7.2 Hz, 2H), 7.27–7.15 (m, 2H), 6.91 (d, *J* = 8.1 Hz, 1H), 6.84 (t, *J* = 7.4 Hz, 1H), 3.78 (s, 2H), 3.67 (s, 6H). ^13^C NMR (126 MHz, DMSO) *δ* 171.83 (s), 165.17 (s), 157.65 (s), 156.14 (s), 151.02 (s), 137.33(s), 137.26 (s), 137.19 (s), 131.44 (s), 130.70 (s), 128.64 (s), 128.39 (s), 128.08 (s), 124.89 (s), 124.51 (s), 121.75 (s), 119.15 (s), 116.62 (s), 115.99 (s), 38.17 (s). MS (ESI) *m*/*z* 503.0 [M + H]^+^; HRMS (ESI) *m*/*z* 503.0269 [M + H]^+^ (calcd for 503.0271, C_22_H_20_BrN_2_O_5_S).

(*E*)-*1*-(*4*-(*6*-*bromo*-*1*,*1*-*dioxidobenzo*[*b*]*thiophene*-*2*-*carbonyl*)*piperazin*-*1*-*yl*)-*3*-(*pyridin*-*2*-*yl*)*prop*-*2*-*en*-*1*-*one (**6q***) Yield 67%, white solid; m.p. 97–99 °C; 3422, 2924, 2853, 1641, 1513, 1459, 1314, 1261, 1218, 1153, 1111, 991, 822, 548 cm^−1^; ^1^H NMR (500 MHz, DMSO) *δ* 8.62 (d, *J* = 3.5 Hz, 1H), 8.31 (s, 1H), 7.98 (dd, *J* = 8.0, 1.7 Hz, 1H), 7.89–7.81 (m, 2H), 7.74 (d, *J* = 7.7 Hz, 1H), 7.65 (d, *J* = 8.1 Hz, 1H), 7.52 (d, *J* = 14.8 Hz, 2H), 7.37 (dd, *J* = 7.1, 4.9 Hz, 1H), 3.77 (s, 2H), 3.67 (s, 6H). ^13^C NMR (126 MHz, DMSO) δ 164.37 (s), 149.68 (s), 149.10 (s), 137.00 (s), 136.05(s), 128.66 (s), 125.81 (s), 124.05 (s), 119.70 (s), 99.43 (s), 72.08 (s), 69.36 (s), 38.17 (s). MS (ESI) *m*/*z* 488.0 [M + H]^+^; HRMS (ESI) *m*/*z* 488.0276 [M + H]^+^ (calcd for 488.0274, C_21_H_19_BrN_3_O_4_S).

(*E*)-*1*-(*4*-(*6*-*bromo*-*1*,*1*-*dioxidobenzo*[*b*]*thiophene*-*2*-*carbonyl*)*piperazin*-*1*-*yl*)-*3*-(*4*-*bromophenyl*)*prop*-*2*-*en*-*1*-*one (**6r***) Yield 92%, white solid; m.p. 215–217 °C; 3425, 1646, 1603, 1433, 1316, 1154, 997, 815, 587, 549 cm^−1^; ^1^H NMR (500 MHz, DMSO) *δ* 8.34 (s, 1H), 8.00 (dd, *J* = 8.0, 1.3 Hz, 1H), 7.87 (s, 1H), 7.72 (d, *J* = 7.5 Hz, 3H), 7.65 (dd, *J* = 16.6, 8.1 Hz, 3H), 7.52 (d, *J* = 15.4 Hz, 1H), 7.36 (t, *J* = 11.2 Hz, 1H), 3.83 (s, 2H), 3.67 (s, 6H). ^13^C NMR (126 MHz, DMSO) *δ* 164.45 (s), 161.64 (s), 157.65 (s), 153.24 (s), 140.45 (s), 137.72 (s), 137.20 (s), 131.61 (s), 129.93 (s), 128.63 (s), 128.38 (s), 124.90 (s), 124.51 (s), 123.46 (s), 122.75 (s), 122.26 (s), 118.94 (s), 38.16 (s). MS (ESI) *m*/*z* 564.9 [M + H]^+^; HRMS (ESI) *m*/*z* 566.9404 [M + H]^+^ (calcd for 564.9427, C_22_H_19_Br_2_N_2_O_4_S).

(*E*)-*1*-(*4*-(*6*-*bromo*-*1*,*1*-*dioxidobenzo*[*b*]*thiophene*-*2*-*carbonyl*)*piperazin*-*1*-*yl*)-*3*-(*4*-*chlorophenyl*)*prop*-*2*-*en*-*1*-*one (**6s***) Yield 92%, white solid; m.p. 225–227 °C; 3421, 1645, 1602, 1491, 1432, 1315, 1222, 1153, 1074, 1153, 1074, 995, 817, 587, 548 cm^−1^; ^1^H NMR (500 MHz, DMSO) *δ* 8.34 (s, 1H), 8.00 (dd, *J* = 8.0, 1.7 Hz, 1H), 7.87 (s, 1H), 7.79 (d, *J* = 8.0 Hz, 2H), 7.67 (d, *J* = 8.0 Hz, 1H), 7.54–7.51 (m, 3H), 7.34 (d, *J* = 15.1 Hz, 1H), 3.83 (s, 2H), 3.67 (s, 6H). ^13^C NMR (126 MHz, DMSO) *δ* 164.46 (s), 160.14 (s), 157.66 (s), 142.96 (s), 140.38 (s), 137.21 (s), 134.00 (s), 132.02 (s), 131.50 (s), 129.69 (s), 128.69 (s), 128.64 (s), 128.39 (s), 127.97 (s), 124.90 (s), 124.52 (s), 118.87 (s), 39.02 (s). MS (ESI) *m*/*z* 523.0 [M + H]^+^; HRMS (ESI) *m*/*z* 522.9014 [M + H]^+^ (calcd for 522.9911, C_22_H_19_BrClN_2_O_4_S).

(*E*)-*1*-(*4*-(*6*-*bromo*-*1*,*1*-*dioxidobenzo*[*b*]*thiophene*-*2*-*carbonyl*)*piperazin*-*1*-*yl*)-*3*-(*3*-*hydroxy*-*4*-*methoxyphenyl*)*prop*-*2*-*en*-*1*-*one (**6t***) Yield 64%, white solid; m.p.123–125 °C; 3397, 2923, 1641, 1514, 1432, 1311, 1155, 1063, 993, 842, 725, 557 cm^−1^; ^1^H NMR (500 MHz, DMSO) δ 9.43 (s, 1H), 7.96–7.95 (m, 2H), 7.92–7.91 (m, 2H), 7.78 (s, 1H), 7.46 (d, *J* = 15.2 Hz, 1H), 7.31 (s, 1H), 7.14 (d, *J* = 8.1 Hz, 1H), 6.78 (d, *J* = 8.1 Hz, 1H), 3.87–3.58 (m, 11H). ^13^C NMR (126 MHz, DMSO) *δ* 166.06 (s), 164.98 (s), 148.55 (s), 148.40 (s), 147.80 (s), 142.57 (s), 142.11 (s), 137.25 (s), 137.16 (s), 134.90 (s), 134.51 (s), 134.32 (s), 122.54 (s), 122.43 (s), 115.43 (s), 114.48 (s), 114.21 (s), 114.15 (s), 111.27 (s), 109.09 (s), 59.65 (s), 55.77 (s), 39.02 (s). MS (ESI) *m*/*z* 533.0 [M + H]^+^; HRMS (ESI) *m*/*z* 533.0377 [M + H]^+^ (calcd for 533.0376, C_23_H_22_BrN_2_O_6_S).

(*E*)-*1*-(*4*-(*6*-*bromo*-*1*,*1*-*dioxidobenzo*[*b*]*thiophene*-*2*-*carbonyl*)*piperazin*-*1*-*yl*)-*3*-(*4*-*hydroxy*-*3*-*methoxyphenyl*)*prop*-*2*-*en*-*1*-*one (**6u***) Yield 76%, white solid; m.p. 201–203 °C; 3423, 1638, 1578, 1511, 1432, 1318, 1261, 1227, 1130, 1026, 993, 547 cm^−1^; ^1^H NMR (500 MHz, DMSO) *δ* 9.06 (s, 1H), 8.34 (s, 1H), 8.00 (dd, *J* = 8.0, 1.4 Hz, 1H), 7.86 (s, 1H), 7.66 (d, *J* = 8.1 Hz, 1H), 7.41 (d, *J* = 15.2 Hz, 1H), 7.19 (s, 1H), 7.13 (d, *J* = 8.2 Hz, 1H), 7.05 (d, *J* = 15.2 Hz, 1H), 6.96 (d, *J* = 8.3 Hz, 1H), 3.83 (s, 5H), 3.66 (s, 6H). ^13^C NMR (126 MHz, DMSO) *δ* 164.92 (s), 157.64 (s), 149.27 (s), 146.51 (s), 142.13 (s), 137.22 (d, *J* = 6.2 Hz), 131.43 (s), 128.63 (s), 128.39 (s), 127.99 (s), 124.89 (s), 124.51 (s), 120.59 (s), 115.12 (s), 114.29 (s), 111.88 (s), 55.62 (s), 54.79 (s), 39.02 (s). MS (ESI) *m*/*z* 533.0 [M + H]^+^; HRMS (ESI) *m*/*z* 533.0381 [M + H]^+^ (calcd for 533.0376, C_23_H_22_BrN_2_O_6_S).

(*E*)-*1*-(*4*-(*5*-*bromo*-*1*,*1*-*dioxidobenzo*[*b*]*thiophene*-*2*-*carbonyl*)*piperazin*-*1*-*yl*)-*3*-(*4*-*fluorophenyl*)*prop*-*2*-*en*-*1*-*one (**6v***) Yield 92%, white solid; m.p. >250 °C; 3443, 1647, 1601, 1509, 1432, 1320, 1264, 1220, 1157, 1073, 992, 825, 548, 512 cm^−1^; ^1^H NMR (500 MHz, DMSO) *δ* 8.34 (s, 1H), 8.00 (d, *J* = 8.0 Hz, 1H), 7.87 (s, 1H), 7.83 (d, *J* = 7.8 Hz, 2H), 7.67 (d, *J* = 8.0 Hz, 1H), 7.55 (d, *J* = 15.4 Hz, 1H), 7.27 (t, *J* = 8.5 Hz, 3H), 3.83 (s, 2H), 3.67 (s, 6H). ^13^C NMR (126 MHz, DMSO) *δ* 164.58 (s),162.01 (s), 157.66 (s), 148.59 (s), 140.58 (s), 137.75 (s), 137.20 (s), 135.51 (s), 131.48 (s), 130.18 (d, *J* = 8.4 Hz), 130.15 (s), 128.64 (s), 128.39 (s), 125.53 (s), 124.90 (s), 124.52 (s), 117.88 (s), 115.70 (s), 115.61 (d, *J* = 21.6 Hz). MS (ESI) *m*/*z* 505.0 [M + H]^+^; HRMS (ESI) *m*/*z* 505.0230 [M + H]^+^ (calcd for 505.0227, C_22_H_19_BrFN_2_O_4_S).

### Docking

The computational docking program AutoDock4.2 was used to dock our designed small molecules to predict their binding modes and approximate binding free energies to the STAT3 SH2 dimerization sites. Briefly, compounds were docked using the Lamarckian Genetic Algorithm. The ligand and macromolecule were prepared using Schrödinger software. Gasteiger charges were assigned to the ligands by Auto Dock Tools. Then, AutoGrid maps were precomputed for all atom types in the ligand set. After 10 million energy evaluations were completed, the root-mean-square deviation threshold was set as 1.5 Å and all the resulting conformations of the ligands in the binding pocket of the macromolecule were clustered. Low energy clusters were identified and binding energies were evaluated.

### Maintenance of cell line cultures and cell viability assays

All cell lines were purchased from Cell Bank of Shanghai Institute of Biochemistry and Cell Biology, Chinese Academy of Sciences (Shanghai, China). Human breast carcinoma cells (MCF-7 and MCF-7/DOX), breast epithelial cells (MCF-10A) and normal human liver cells (LO2) were maintained in RPMI-1640 medium. All cells were supplemented with 12% fetal bovine serum containing 50 μg/ml penicillin and 50 μg/ml streptomycin. Cells were grown to 80% confluency in a tissue culture flask at 37 °C in a humidified atmosphere containing 5% CO_2_, and then were trypsinized with 1 × Trypsin-Versene and split. The MCF-7/DOX cell line we used was established from its parental cell line MCF-7 cell by gradually increasing the concentration of DOX to which the cells were exposed in a stepwise manner over a period of 8 months. A 5 nM to 100 nM range of DOX concentrations was added to MCF-7 cells, after which the cells were maintained in culture medium containing 100 nM DOX and displayed a 66.02-fold resistance to DOX compared with the corresponding parental, DOX-sensitive cells. The cells were incubated in drug-free medium for at least 1 week before use.

Cells (MCF-7, MCF-7/DOX, MCF-10A, LO2) were seeded in 96-well plates at a density of 3,000–6,000 cells per well. The cells were incubated overnight (16 h) in a humidified 5% CO_2_ incubator at 37 °C. After media removal, different concentrations of test compounds were added in triplicate to the plates in 200 μL of fresh media and were incubated with the cells at 37 °C for 48 h. The percentage of DMSO in the medium did not exceed 0.1%. Cell viability was evaluated using 3-(4, 5-dimethylthiazolyl)-2,5-diphenyltetrazoliumbromide (MTT). The absorbance was read by an ELISA reader (SpectraMax Plus384, Molecular Devices, Sunnyvale, CA) at a test wavelength of 570 nm and a reference wavelength of 630 nm. Cell viability was calculated using the following formula:


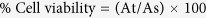


At and As denote the absorbance of the test substance and solvent control, respectively.

### Western blot assays

MCF-7 or MCF-7/DOX cells were incubated with various concentrations of **6b** for 24 h. After trypsinization, cells were treated with 1× RIPA lysis buffer (50 mM Tris–HCl, pH 7.4, 150 mM NaCl, 0.25% deoxycholic acid, 1% NP-40, 1 mM EDTA and protease inhibitors) (Amresco, Solon, USA) to extract the total proteins. An aliquot of proteins from the total cell lysates (30 to 60 μg/lane) was separated by sodium dodecyl sulfate (10%) polyacrylamide gel electrophoresis (SDS–PAGE, Bio-Rad Laboratories, Hercules, CA), wet-transferred to NC membrane (Bio-Rad Laboratories, Hercules, CA) and blotted with primary antibodies specific for STAT3, p-STAT3, p-Jak2, Jak2, p-Src, Src, Erk, P-Erk, PARP, cleaved PARP, Bcl-2, Bax, Cyclin D1, P-gp and GAPDH. Bound immuno-complexes were detected using ChemiDOC™ XRS + system (Bio-Rad Laboratories, Hercules, CA).

### Quantitative real-time RT-PCR

Levels of mRNA expression were analyzed with the RT-PCR assay, with total RNA isolated from MCF-7 or MCF-7/DOX cells using an EASYspin Plus tissue/cell RNA extraction kit (Aidlab Biotechnologies Co. Ltd). RNA was quantified by measuring absorption at 260 nm and 1 μg of RNA was reverse transcribed to cDNA using the Transcriptor First Strand cDNA Synthesis Kit (Roche Diagnostics, Basel, Switzerland). Thermal cycling conditions included 95 °C initial denaturation for 5 min, followed by 40 cycles of denaturation (10 s at 95 °C), annealing (15 s at 60 °C) and extension (15 s at 72 °C with a single fluorescence measurement), a melt curve program (60–95 °C with a 0.11 °C/s heat increase and continuous fluorescence measurement) and a cooling step to 40 °C. The Δ cycle threshold method was used for the calculation of relative differences in mRNA abundance with a LightCycler 480 (Roche Molecular Biochemicals, Mannheim, Germany). The data were normalized to the expression of GAPDH. The results were expressed as fold-changes. The RT-PCR primers that were used in this study are listed in the supporting information.

### IL-6 induced STAT3 phosphorylation and IFN-γ induced STAT1 phosphorylation

MCF-7 or MCF-7/DOX cells were seeded in 6-well plates and allowed to adhere overnight. The following day, the cells were serum-starved. The cells were then left untreated or were treated with **6b** (0.5–4 μM). After 6 h, the untreated and **6b**-treated cells were stimulated with IL-6 (50 ng/ml). The cells were harvested after 30 min and analyzed by western blot.

### Immunofluorescence staining

The basic protocol for the analysis of p-STAT3 subcellular location has been previously described^39^. Briefly, MCF-7 cells were starved overnight, followed by incubation with **6b** for 6 h. Cells were then stimulated with 50 ng/ml IL-6 for 30 min before being harvested in Tris-buffered saline (TBS) containing 1 mM sodium orthovanadate. Cells were transferred to polylysine-coated slides by cytospin and air dried for 20 min at room temperature. After being fixed in 2% of paraformaldehyde for 10 min and permeabilized in 0.1% Triton X-100 for 5 min, cells were blocked in TBS containing 5% bovine serum albumin for 1 h at room temperature before being stained with p-STAT3 (Tyr705) overnight at 4 °C. Then, cells were gently washed and incubated with FITC–conjugated, goat, anti-rabbit IgG (Beyotime Biotechnology Ltd). Finally, slides were incubated with 5 μg/ml of 4′,6-diamidino-2-phenylindole (DAPI, Beyotime) for 5 min. The subcellular location and relative abundance of p-STAT3 were immediately analyzed with an ImageXpress Micro Confocal analysis.

### Electrophoretic mobility shift assay (EMSA) of DNA-binding activity

MCF-7 cells were cultured overnight and then treated with compound **6b** in fresh regular growth medium for 24 h. The cells were harvested and nuclear extract preparations and EMSA analysis were carried out as previously described^3^. The ^32^P-labeled oligonucleotide, hSIE probe, was used to bind STAT3.

### Flow cytometry analysis of apoptotic cells

MCF-7, MCF-7/DOX or MCF-10A cells, at a density of 2.5 × 10^5^ cells per well, were cultured in regular growth medium in 6-well plates for 24 h and treated in duplicate with different concentrations of **6b** or curcumin for 24 h. The cells were harvested, washed and stained with 5 μL of Annexin V-APC and 5 μL of 7-AAD at room temperature for 15 min. Cells were then analyzed by flow cytometry (488 nm excitation and 600 nm emission filters) using a BD FACSCalibur flow cytometer (Becton & Dickinson Company, Franklin Lakes, NJ).

### Flow cytometry analysis of the cell cycle

MCF-7 or MCF-7/DOX cells were plated in 6-well culture plates (1.5 × 10^5^ cells per well). After incubation overnight, various concentrations of **6b** or curcumin were added and cells were incubated for 24 h. Cells were harvested, washed with cold PBS, and incubated with pre-cooled 70% ethanol for a minimum of 4 h at 4 °C to fix them. The cells were then centrifuged, carefully aspirated from the supernatant, and re-suspended in a propidium iodide (final concentration, 40 μg/ml) and RNase A (final concentration, 100 μg/ml) solution at a final cell density of 0.5 × 10^6^ cells/ml. The suspension was incubated at 37 °C for 30 min prior to analysis by flow cytometry.

### Detection of ROS using flow cytometry

MCF-7 or MCF-7/DOX cells were seeded at a density of 3 × 10^5^ cells per well of a 6-well plate and treated with various concentrations of **6b** (0, 1, 2, 4 μM) or 4 μM curcumin for 24 h. DCFH-DA was dissolved in serum-free medium and diluted to a final concentration of 10 μM. After treatment with **6b**, the growth media was replaced with serum-free medium containing the probe. After incubation for 20 min at 37 °C, cells were washed with serum-free medium twice, digested by trypsin and resuspended in pre-warmed PBS buffer. The samples were then subjected to a flow cytometry assay using a BD FACSCalibur flow cytometer (Becton & Dickinson Company, Franklin Lakes, NJ).

### Colony survival assay

MCF-7 or MCF-7/DOX cells were cultured a 6-well plate at a density of 800–1,000 cells/well with regular growth medium. Cells were treated for 24 h with the vehicle and **6b** at 0.15–1 μM on the following day. Cells were allowed to grow for 10–14 d until colonies were visible. Crystal violet solution (Sigma, St. Louis, MO, USA) was used to stain the colonies for 4 hours and colonies were imaged.

### *In vivo* studies

The working solution was prepared in a Cremophor and DMSO mixture (10% Cremophor, 10% DMSO and 80% 1 × PBS) to give a 1 mg/ml solution. Athymic BALB/c nude mice (15–18 g) were injected with human breast tumor MCF-7 cells (3 × 10^6^ cells in a volume of 0.2 ml) into the subcutaneous tissue of the right auxiliary region of the mice. Three days after the tumor cell inoculation, the mice were randomly sorted into three groups with six mice per group. The tumor-bearing mice were either given a daily 10 mg/kg *ip* injection of compound **6b** or the vehicle. The treatment was initiated when the tumor burden of the mice reached approximately 50 mm^3^. The tumor size was measured by caliper three times a week to document tumor growth and calculated by the formula: length × width × width/2, and the body weight was measured and recorded. Therapeutic efficacy was evaluated based on body weight loss, tumor growth inhibition [determined by using calipers and calculated by the formula: tumor inhibit rate (%) = (tumor vol_Con_ − tumor vol_Tre_)/tumor vol_Con_] × 100. The body weight inhibitory ratio, BI (%) was calculated as BI (%) = (1 −M_T_/M_C_) × 100, where M_T_ and M_C_ were the average body weights of the treated and control groups, respectively. On the 22^nd^ day, all mice were killed, and the tumors were segregated, weighed, and stored in −80 °C for later use. The levels of p-STAT3 in tumor tissues prepared from control or mice treated with **6b** were analyzed by western blot assay. Apoptosis was evaluated via the TUNEL assay, and the Ki-67 assay was used to assess cancer cell proliferation.

The experimental mice were cared for and handled strictly according to the requirements of the Animal Ethics Committee of China Pharmaceutical University and the National Institutes of Health (NIH) standard guidelines for the Care and Use of Laboratory Animal. All experimental protocols were approved by the Animal Ethics Committee of China Pharmaceutical University.

### Statistical analyses

All experiments were conducted more than three times. The results were analyzed using GraphPad Prism version 5.0 (GraphPad Software, San Diego, CA, USA) to perform one-way ANOVA. The results are given as the mean ± SD. A p value less than 0.05 was considered statistically significant.

## Additional Information

**How to cite this article**: Zhang, W. *et al*. Discovery of monocarbonyl curcumin-BTP hybrids as STAT3 inhibitors for drug-sensitive and drug-resistant breast cancer therapy. *Sci. Rep.* **7**, 46352; doi: 10.1038/srep46352 (2017).

**Publisher's note:** Springer Nature remains neutral with regard to jurisdictional claims in published maps and institutional affiliations.

## Supplementary Material

Supplementary Information

## Figures and Tables

**Figure 1:**
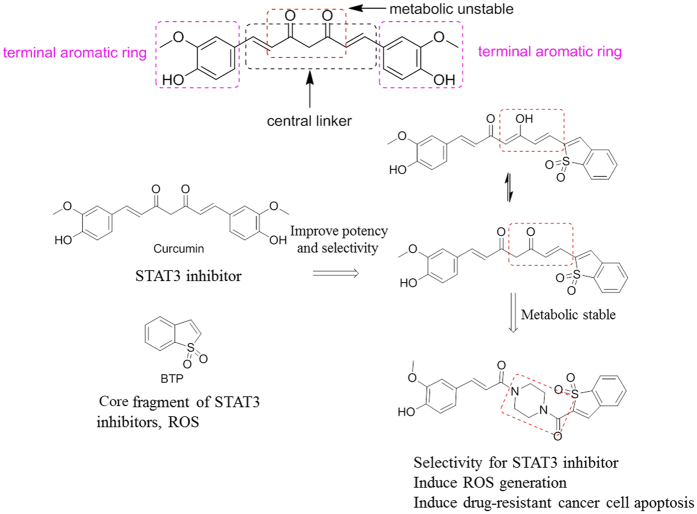
Design strategy of the new curcumin-BTP hybrids.

**Table 1:** Anti-proliferative activity of the designed compounds and the reference compounds, curcumin and DOX.

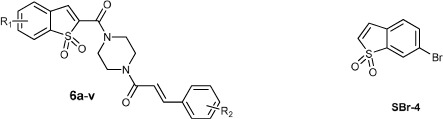				
Compd	R_1_	R_2_	MCF-7	MCF-7/DOX
			IC_50_ ± SD (μM)^a^	
**6a**	5-Br	3,4-(OCH_3_)_2_	0.71 ± 0.14	1.82 ± 0.19
**6b**	5-Br	4-OCH_3_	0.52 ± 0.16	0.40 ± 0.01
****6c****	5-Br	3,4,5-(OCH_3_)_3_	1.23 ± 0.08	0.86 ± 0.13
**6d**	5-Br	3,5-(OCH_3_)_2_-4-OH	2.25 ± 0.35	2.27 ± 0.60
**6e**	5-Br	4-NO_2_	1.18 ± 0.08	1.08 ± 0.14
**6f**	5-Br	Py	1.08 ± 0.06	1.89 ± 0.27
**6g**	5-Br	4-Br	2.08 ± 0.06	1.18 ± 0.07
**6h**	5-Br	4-Cl	2.02 ± 0.20	0.72 ± 0.13
**6i**	5-Br	4-CF_3_	0.67 ± 0.14	0.87 ± 0.12
**6j**	5-Br	3- OH -4-OCH_3_	0.86 ± 0.09	1.00 ± 0.76
**6k**	5-Br	4-F	1.50 ± 0.18	2.44 ± 0.57
**6l**	6-Br	3,4-(OCH_3_)_2_	1.25 ± 0.14	1.77 ± 0.12
**6m**	6-Br	4-OCH_3_	0.99 ± 0.07	0.38 ± 0.01
**6n**	6-Br	3,4,5-(OCH_3_)_3_	2.62 ± 0.13	2.88 ± 0.57
**6o**	6-Br	3,5-(OCH_3_)_2_-4-OH	2.90 ± 0.36	9.49 ± 2.80
****6p****	6-Br	2-OH	4.00 ± 0.91	3.78 ± 0.14
**6q**	6-Br	Py	1.48 ± 0.19	4.42 ± 0.17
**6r**	6-Br	4-Br	1.60 ± 0.13	2.61 ± 0.82
**6s**	6-Br	4-Cl	1.60 ± 0.09	2.23 ± 0.16
**6t**	6-Br	3-OCH_3_-4-OH	3.03 ± 0.22	2.02 ± 0.34
**6u**	6-Br	3- OH -4-OCH_3_	1.44 ± 0.11	0.55 ± 0.01
**6v**	6-Br	4-F	1.45 ± 0.17	2.14 ± 0.27
**5Br-4**			3.92 ± 0.31	3.48 ± 0.57
**curcumin**			37.70 ± 8.20	32.7 ± 4.00
**DOX**			0.98 ± 0.08	64.7 ± 3.80

The inhibitory effects of the compounds on the proliferation of MCF-7 and MCF-7/DOX cell lines were determined by the MTT assay. SD: standard deviation, all experiments were independently performed at least three times.

**Table 2:** Anti-proliferative activity of **6b** or DOX on MCF-10A and LO2 cells.

**Compd**	MCF-10A	LO2
6b	7.72 ± 0.56	8.31 ± 0.41
DOX	0.98 ± 0.17	0.82 ± 0.12

The inhibitory effects of the compounds on the proliferation of MCF-10A and LO2 cell lines were determined by the MTT assay. SD: standard deviation, all experiments were independently performed at least three times.
