# Supplementary material for: Discovery of monocarbonyl curcumin-BTP hybrids as STAT3 inhibitors for drug-sensitive and drug-resistant breast cancer therapy
Source: Sci Rep. 2017 Apr 11;7:46352. doi: 10.1038/srep46352 (PMC5387716; doi:10.1038/srep46352)

Discovery of monocarbonyl curcumin-BTP hybrids as STAT3 inhibitors for drug-sensitive and drug-resistant breast cancer therapy

Wenda Zhang, Jianpeng Guo, Shanshan Li, Ting Ma, Dingqiao Xu, Chao Han, Feiyan Liu, Wenying Yu* & Lingyi Kong*

State Key Laboratory of Natural Medicines, Department of Natural Medicinal Chemistry, China Pharmaceutical University, 24 Tong Jia Xiang, Nanjing 210009, People' s Republic of China

* Corresponding Authors. Tel/Fax: +86-25-83271405; E-mail: cpu_lykong@126.com (Lingyi Kong); ywy@cpu.edu.cn (Wenying Yu).

Contents

Scheme1. Synthesis of the new curcumin-BTP hybrids

S1. The ^{1}H NMR spectrums of representative compounds

S1. Synthesis of the new curcumin-BTP hybrids

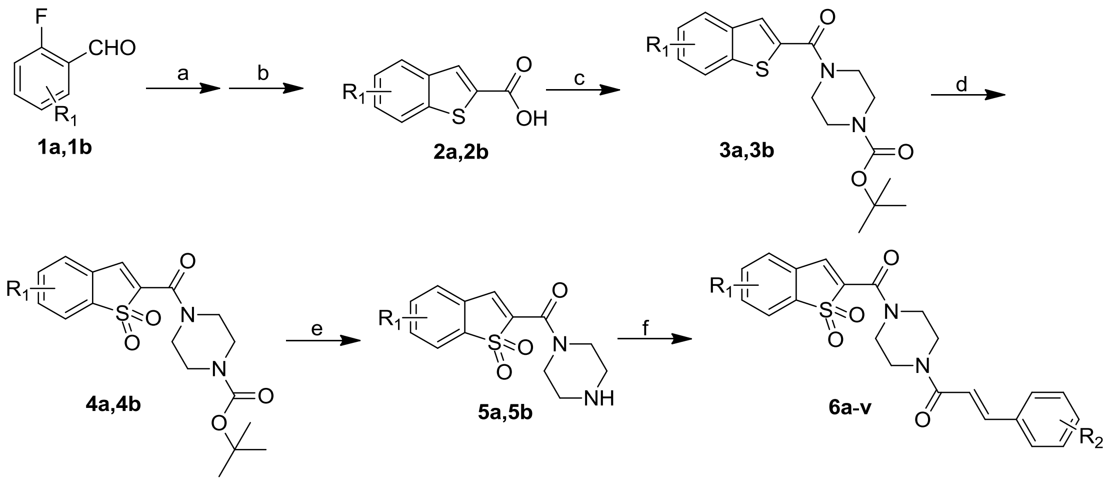

Reagents and conditions: (a) $HSCH_2COOCH_3$, DMF, 60 °C, 16h; (b) 10% KOH, reflux, 3 h, 1 M HCl; (c) SO_2Cl, reflux; Et_3N,overnight; (d) m-CPBA, DCM, reflux, 8h;(e) HCl gas in MeOH, rt, 48 h; (f) HATU, Et3N, DCM, rt, overnight.

Reagents and conditions: (a) $HSCH_2COOCH_3$, DMF, 60 ℃, 16h; (b) 10% KOH, reflux, 3 h, 1 M HCl; (c) SO_2Cl, reflux; Et_3N, DCM, 0 ℃ , overnight; (d) m-CPBA, DCM, reflux, 8h;(e) HCl gas in MeOH, rt, 48 h; (f) HATU, Et_3N, DCM, rt, overnight.

S2. The ^{1}H NMR spectrums of representative compounds

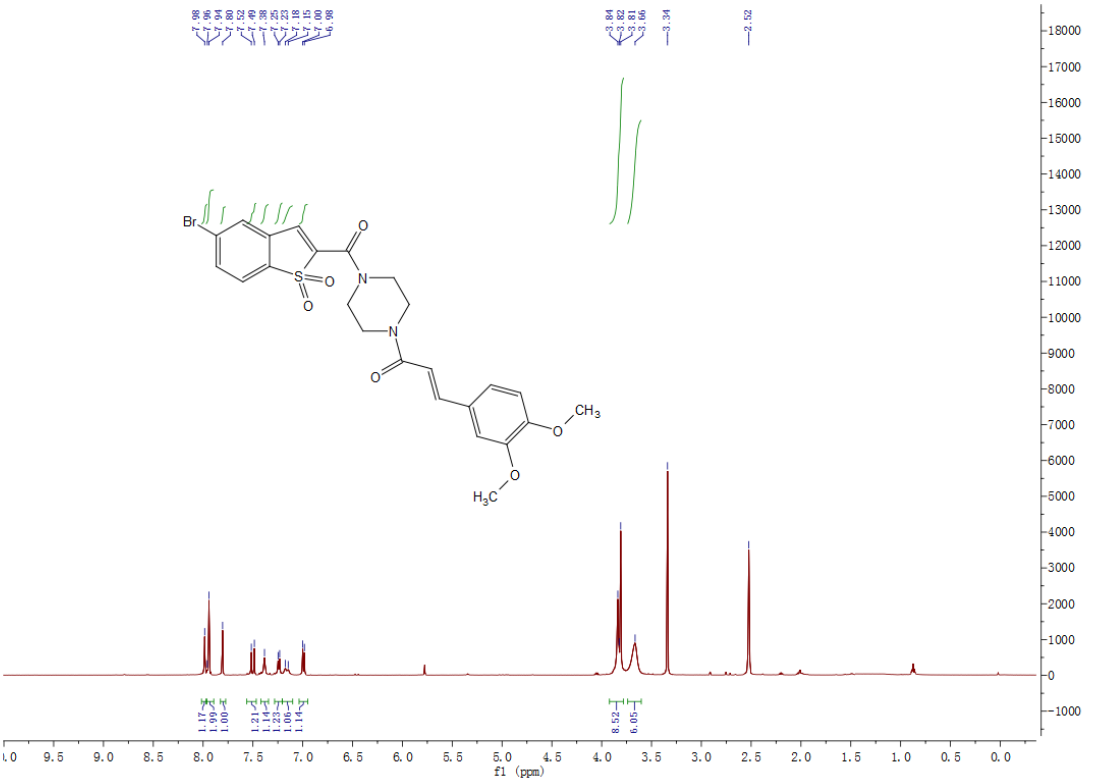

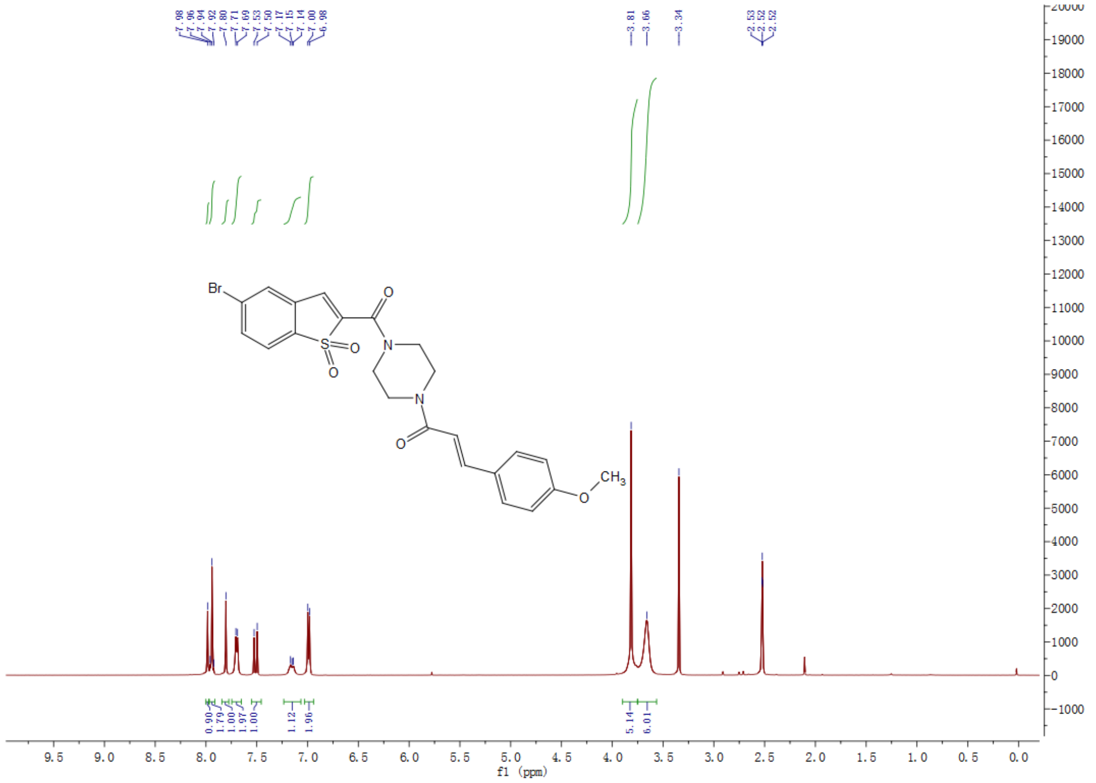

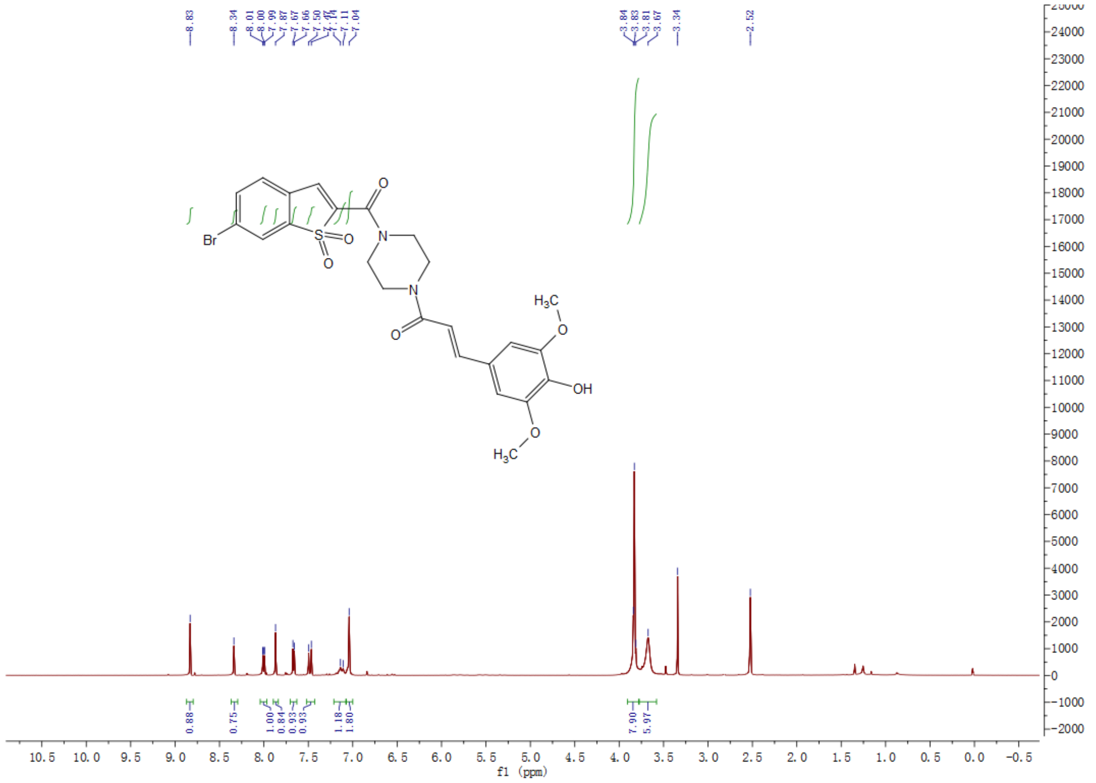

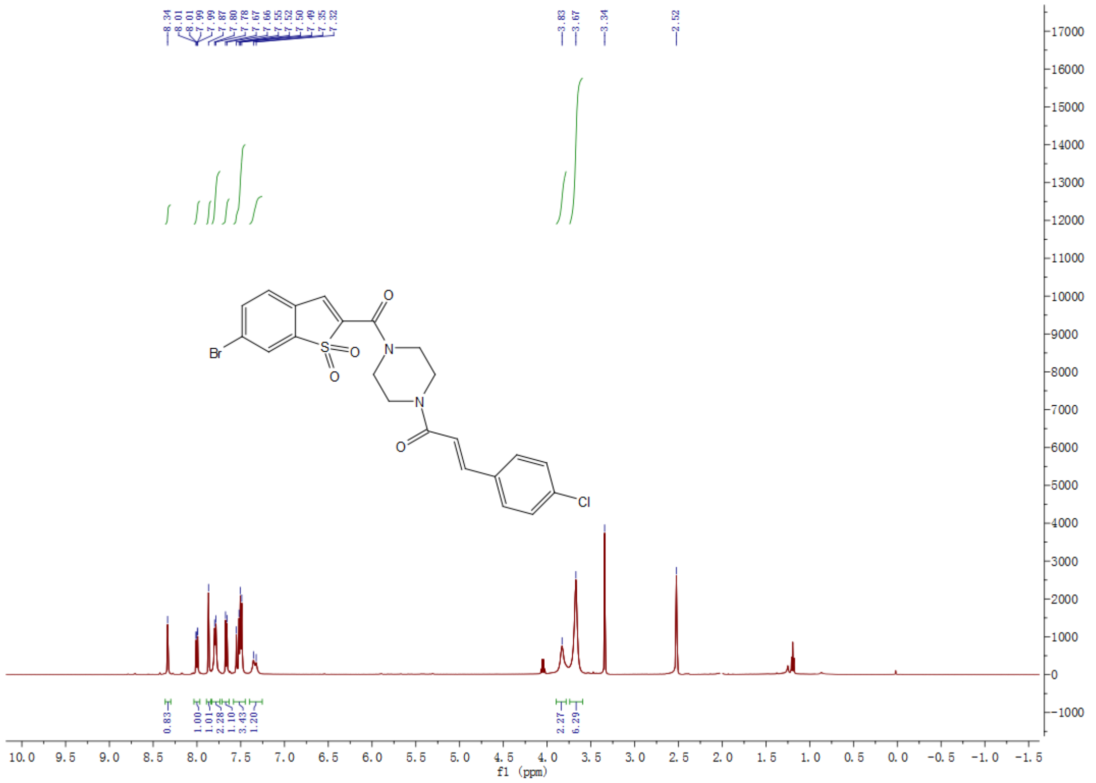

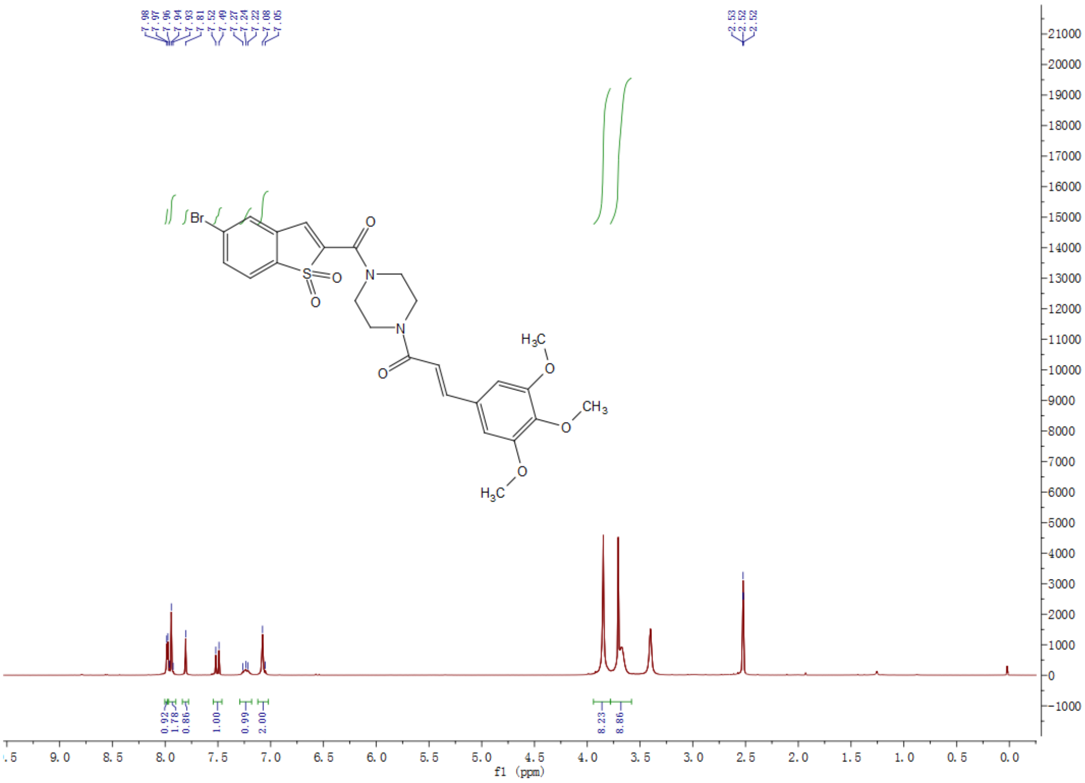

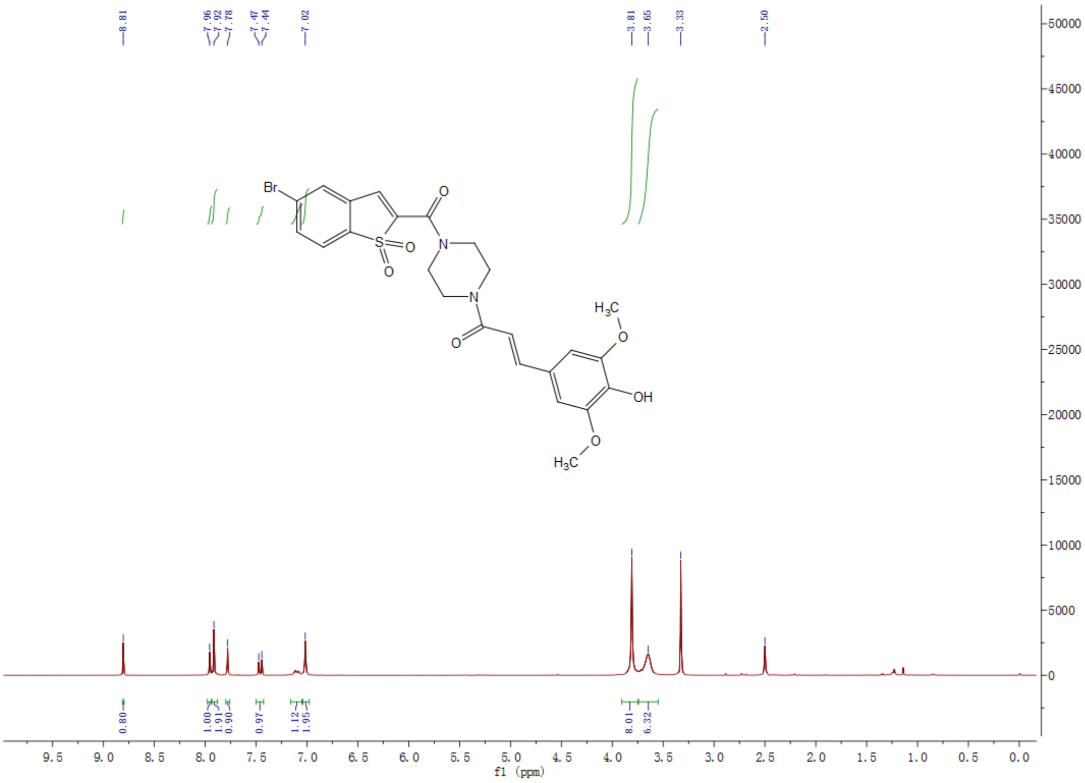

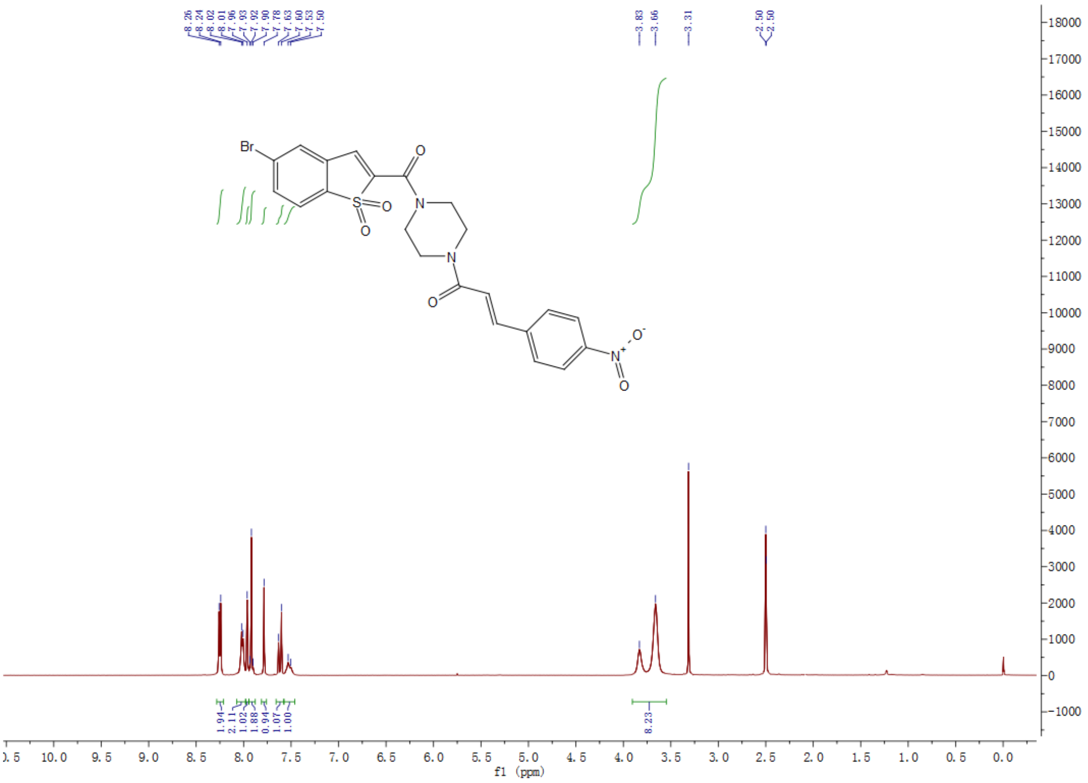
Br
O
S
O
N
N
O
N
O
O
f1 (ppm)

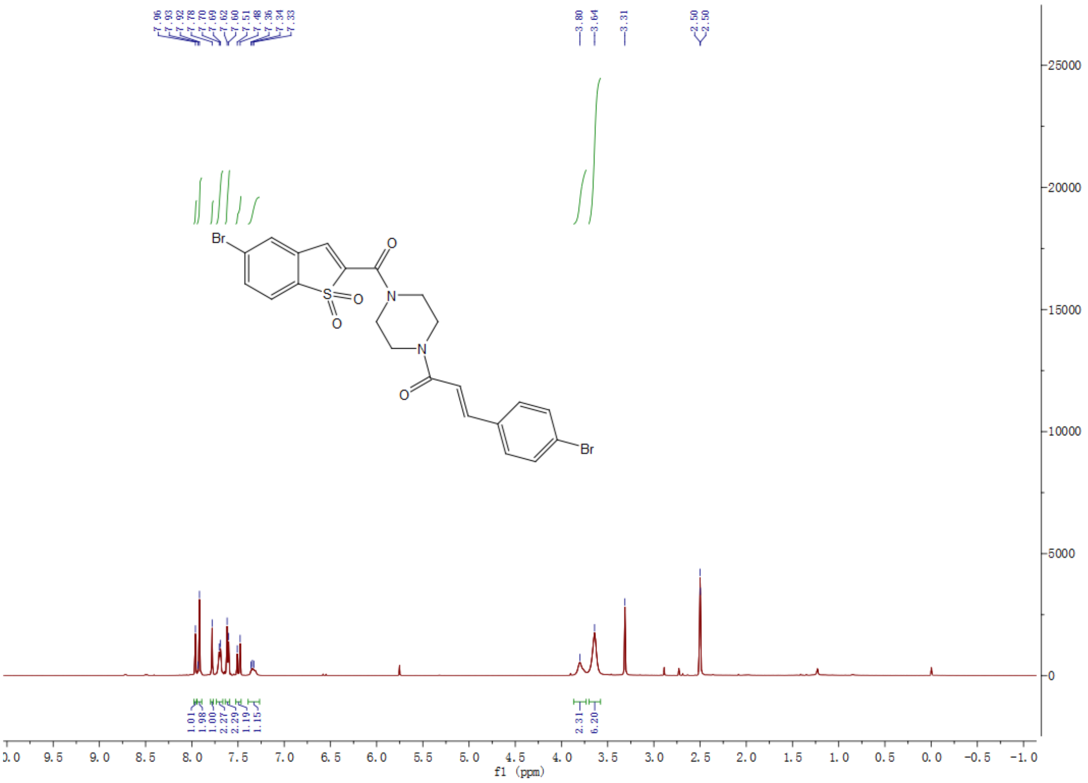
Br
O
S
O
N
N
O
Br
f1 (ppm)

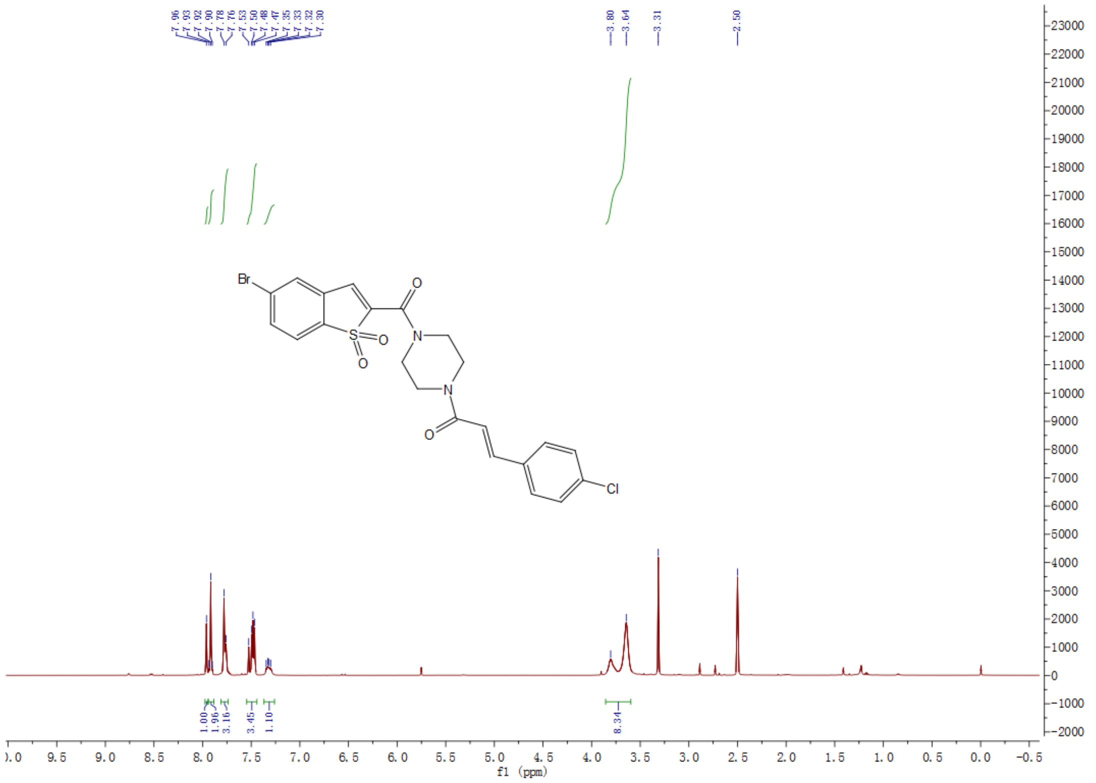
7.96
7.93
7.92
7.90
7.78
7.76
7.53
7.50
7.48
7.47
7.35
7.33
7.32
7.30
3.80
3.64
3.31
2.50
Br
O
S
O
O
N
N
O
Cl
1.00
1.96
3.16
3.45
1.10
8.34
f1 (ppm)

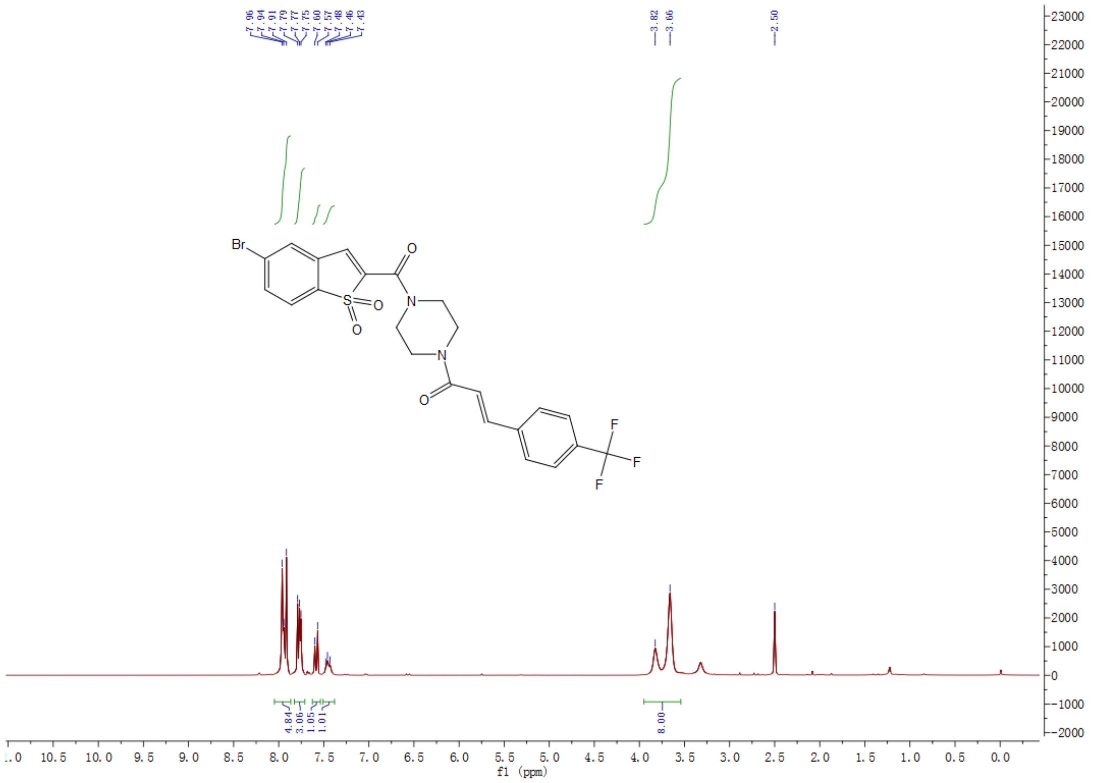
7.96
7.94
7.91
7.79
7.77
7.75
7.60
7.57
7.48
7.46
7.43
3.82
3.66
2.50
Br
O
S
O
O
N
N
O
F
F
F
4.84
3.06
1.05
1.01
8.00
f1 (ppm)

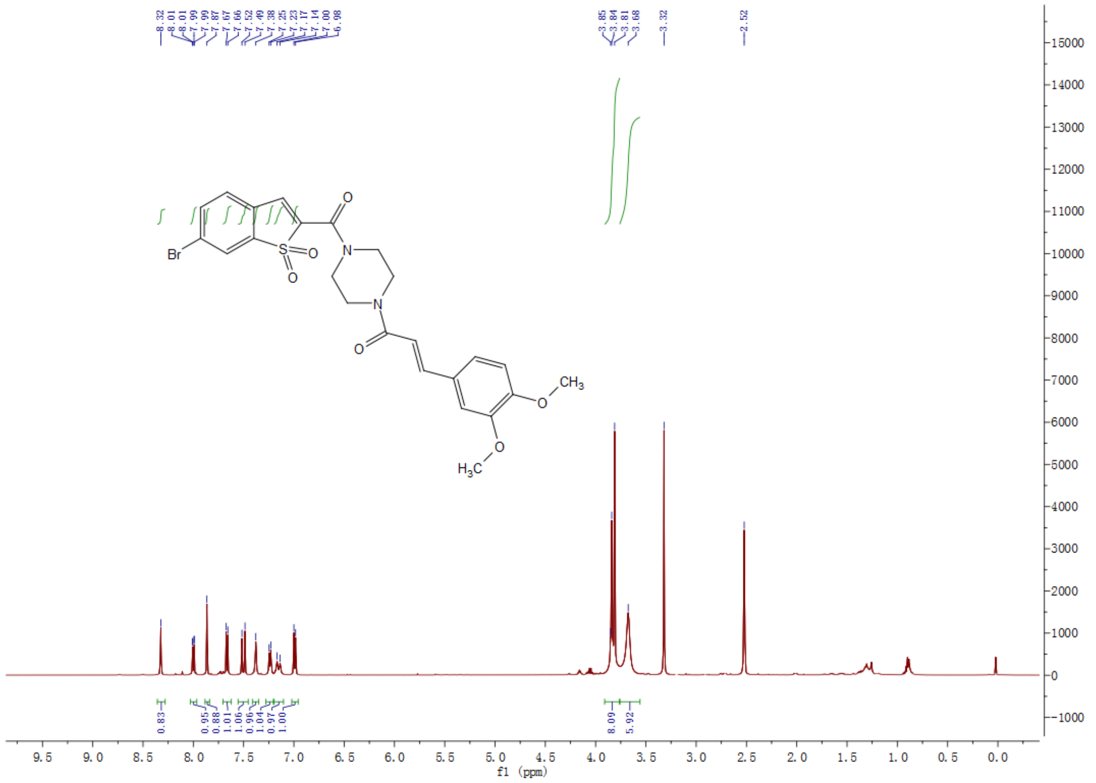

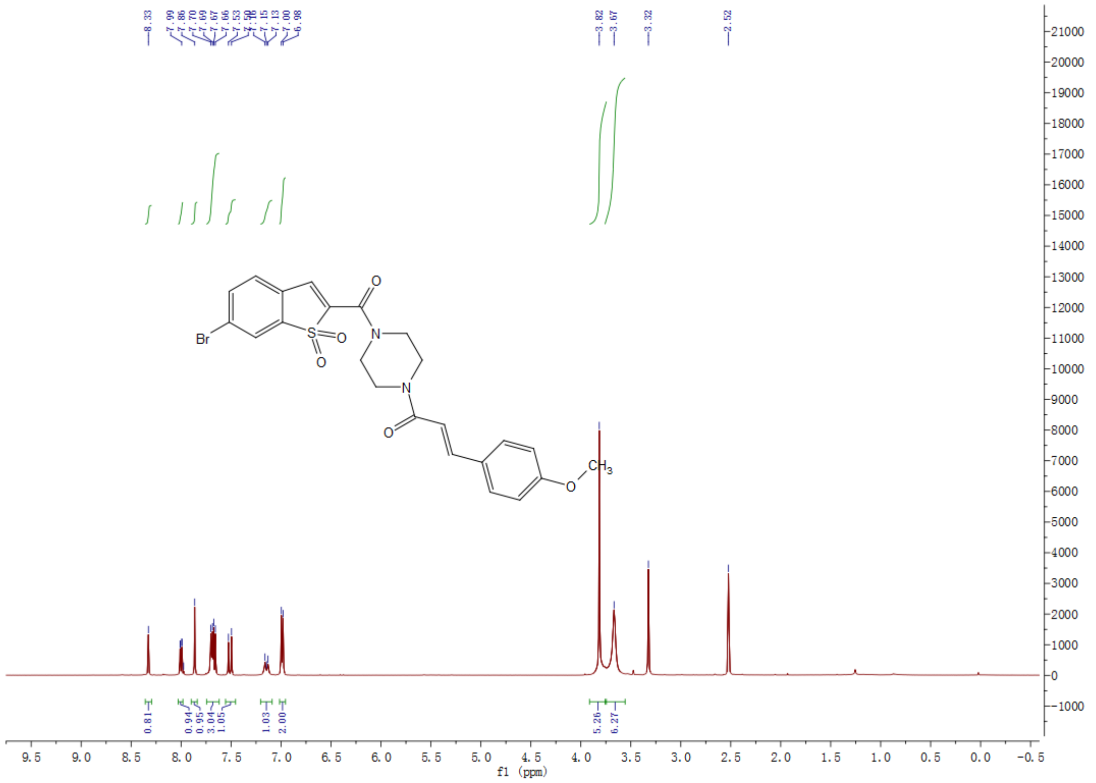

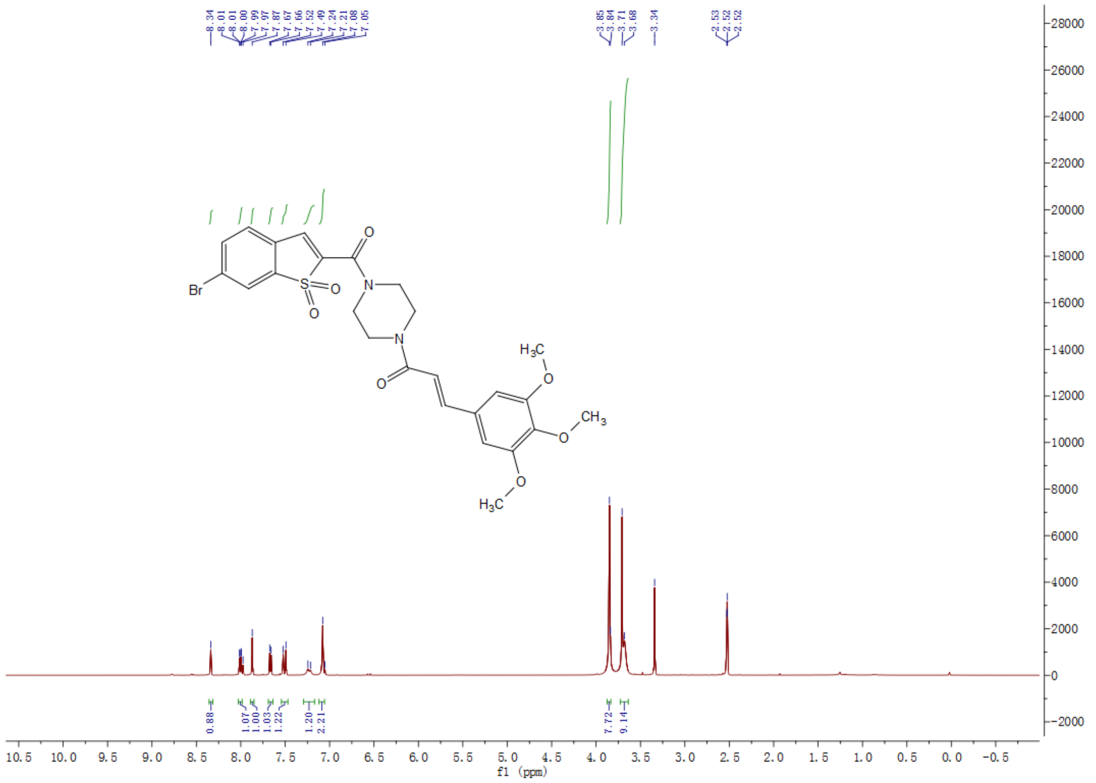

Supplement: Supplementary Information [file srep46352-s1.pdf]
